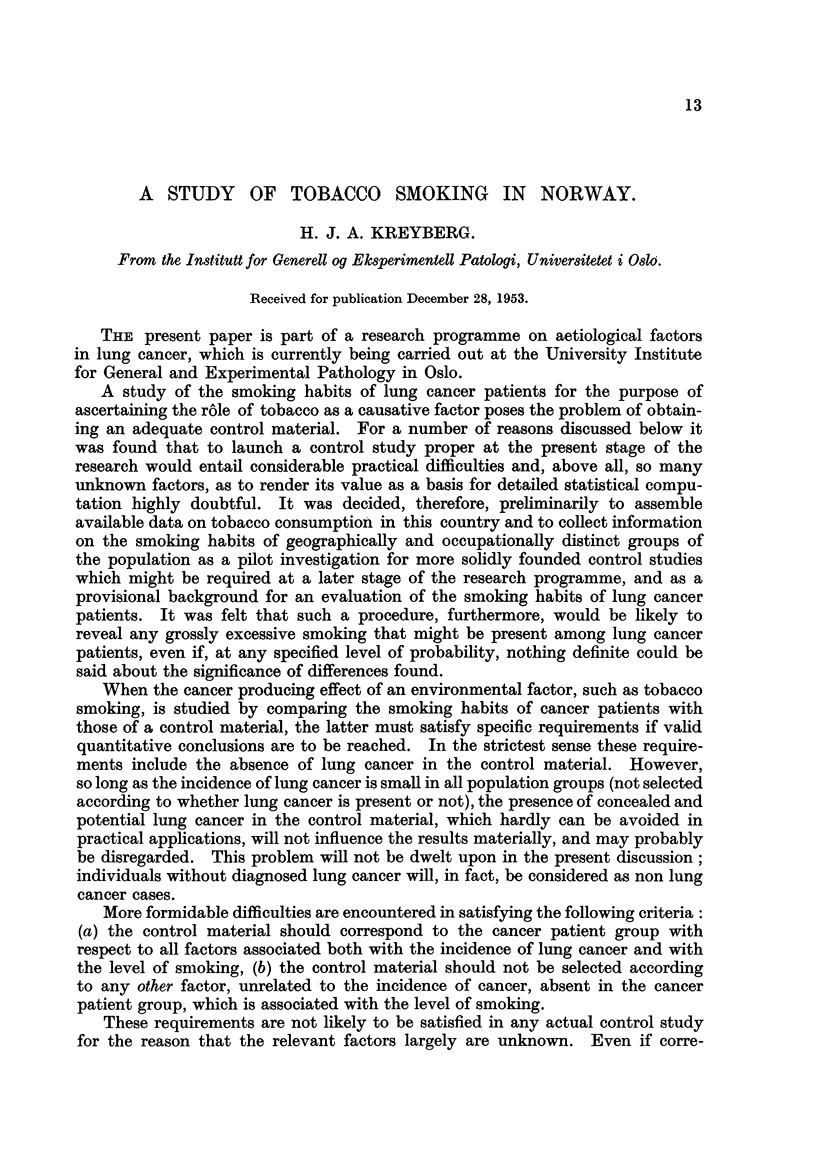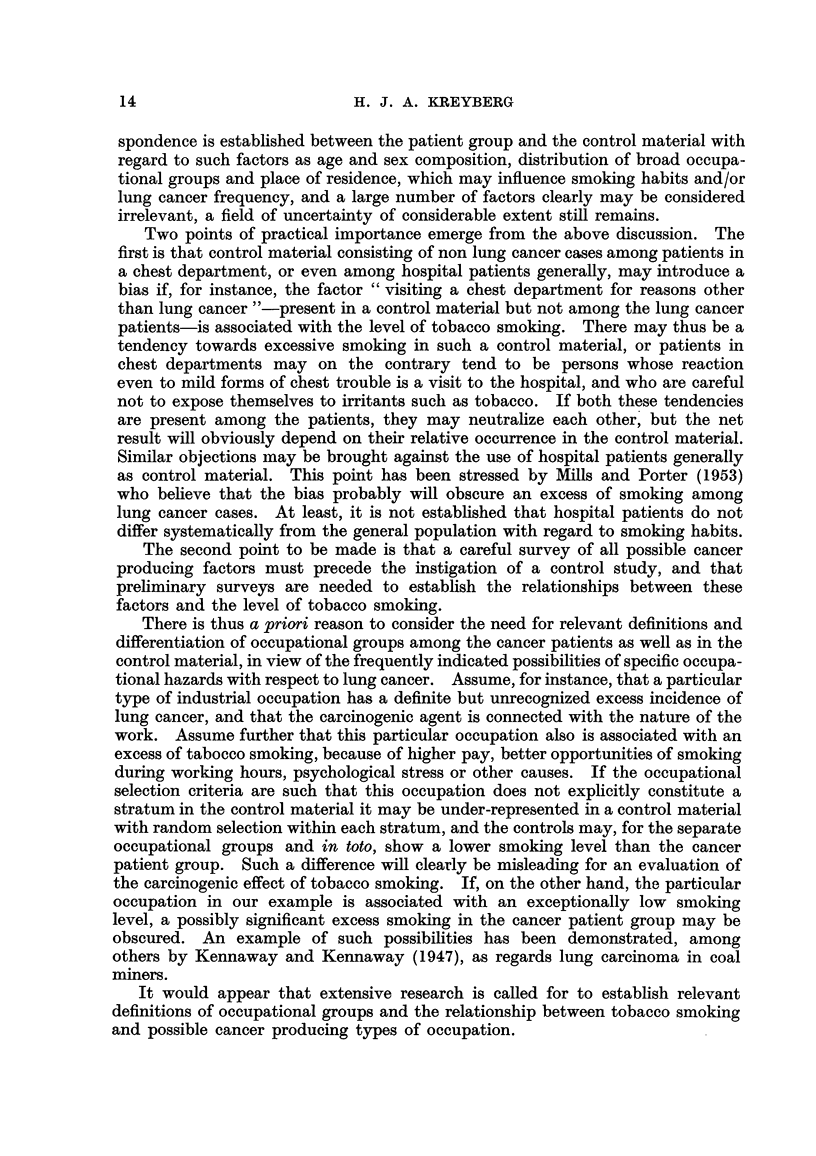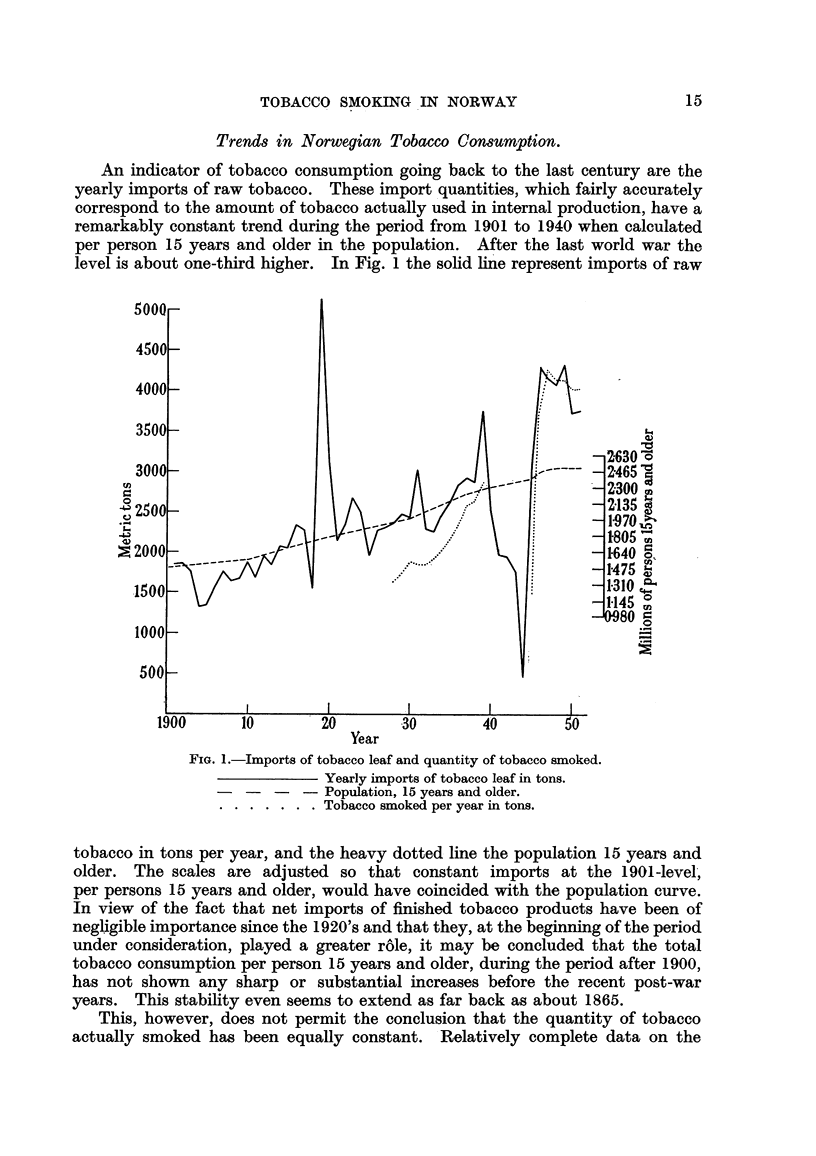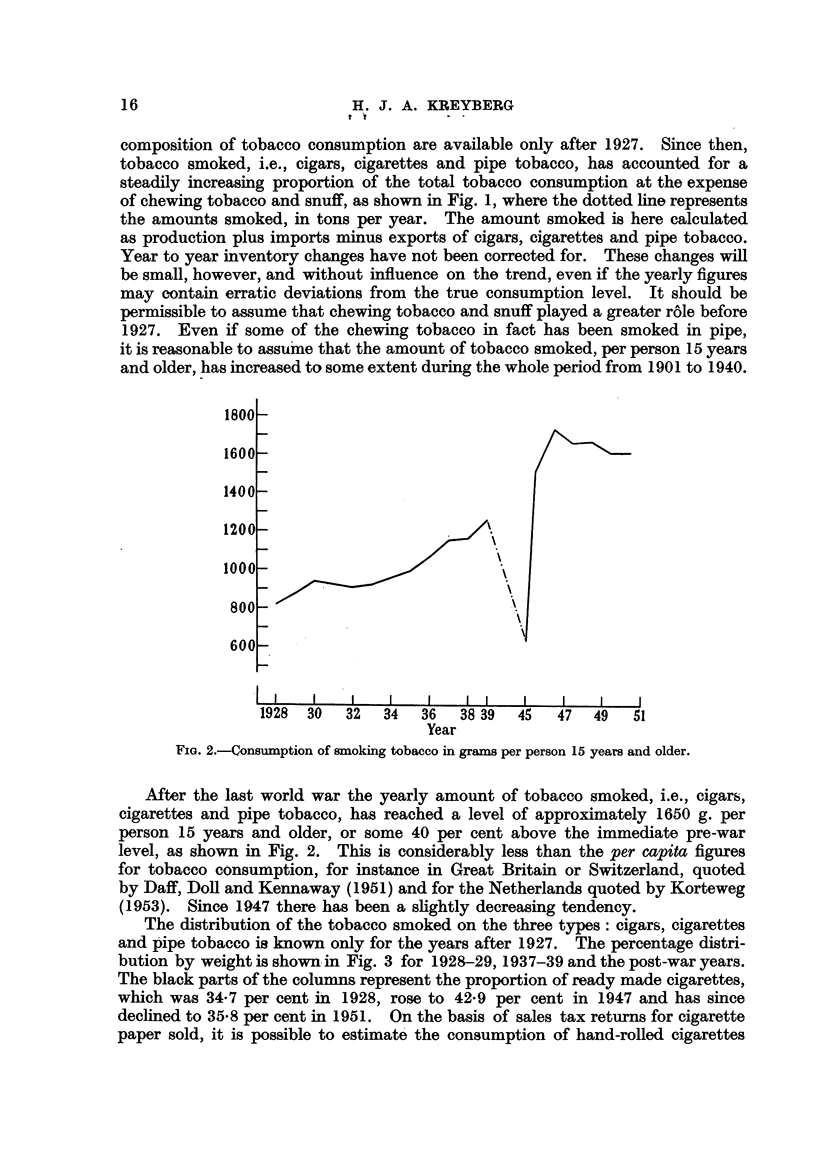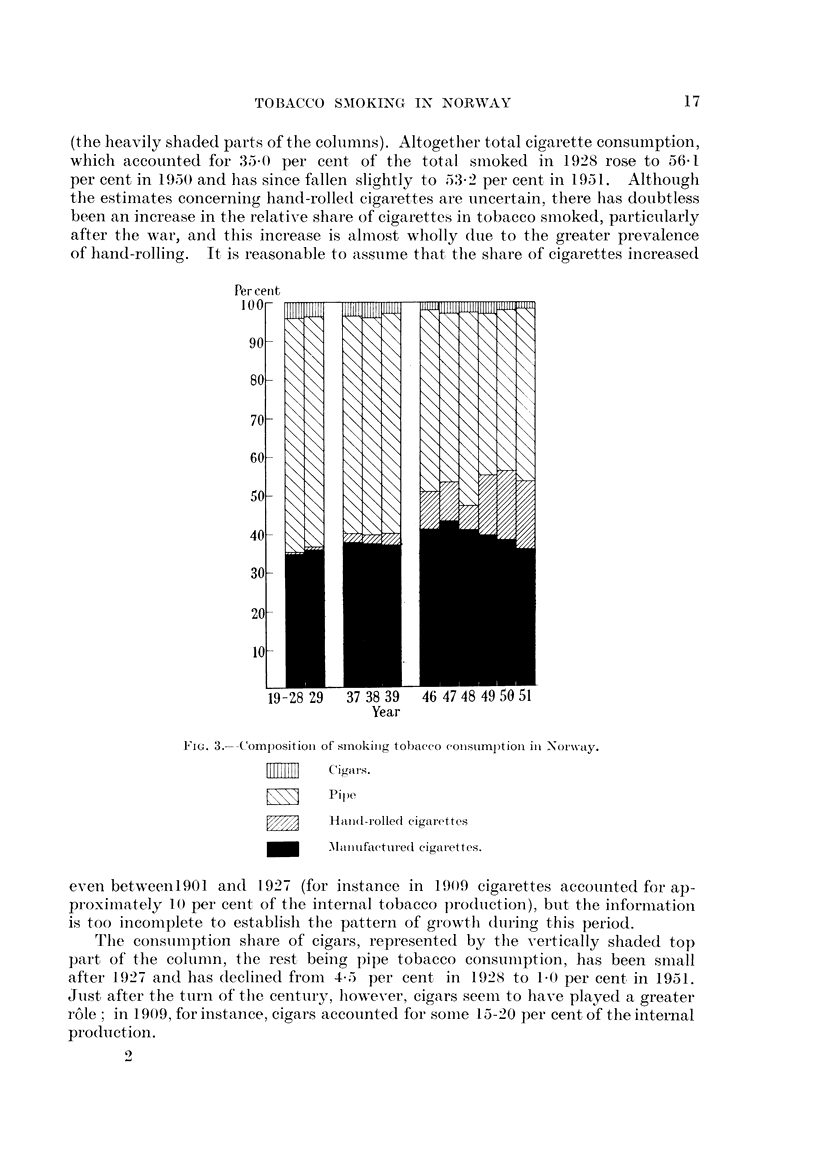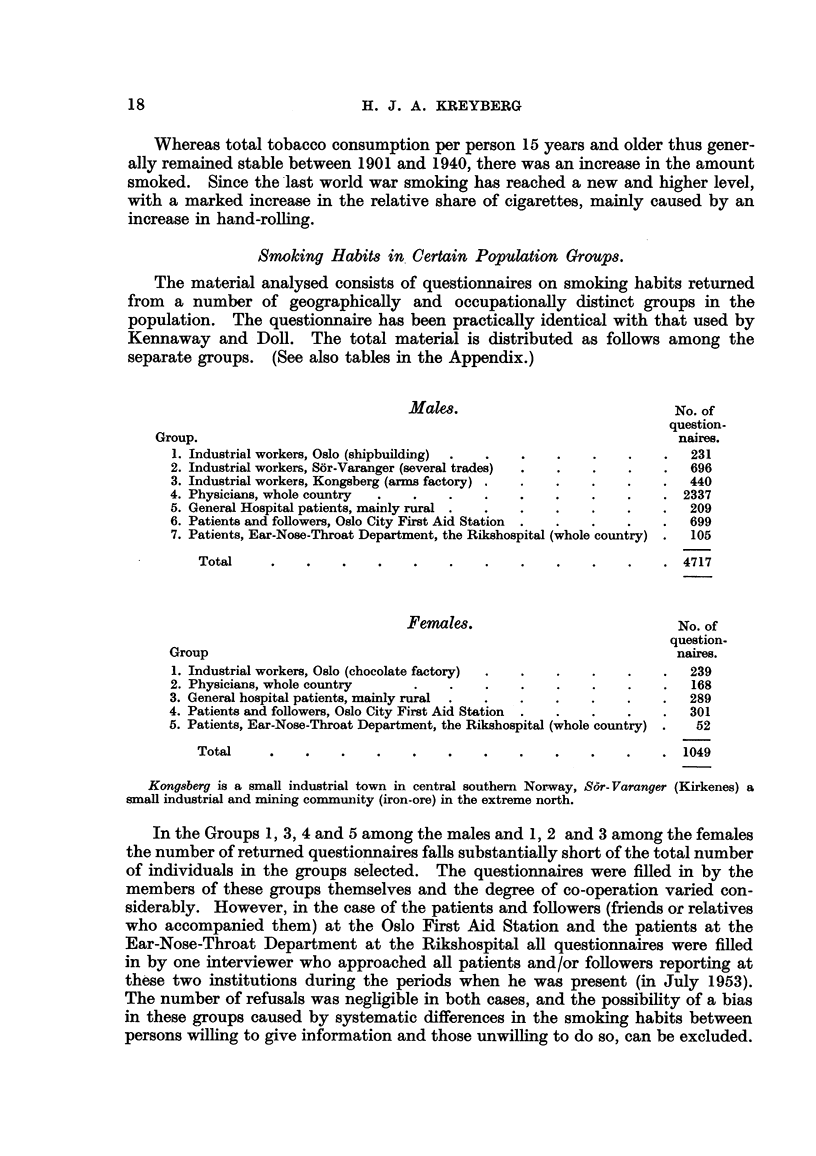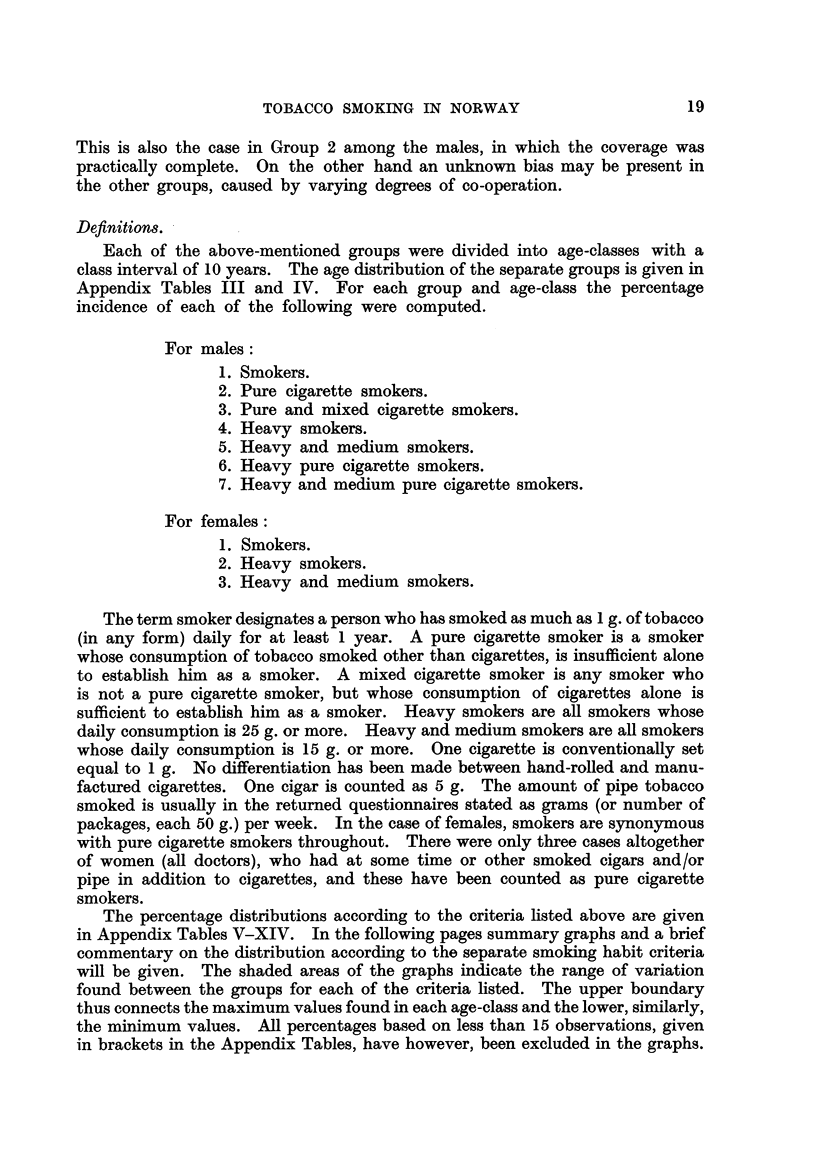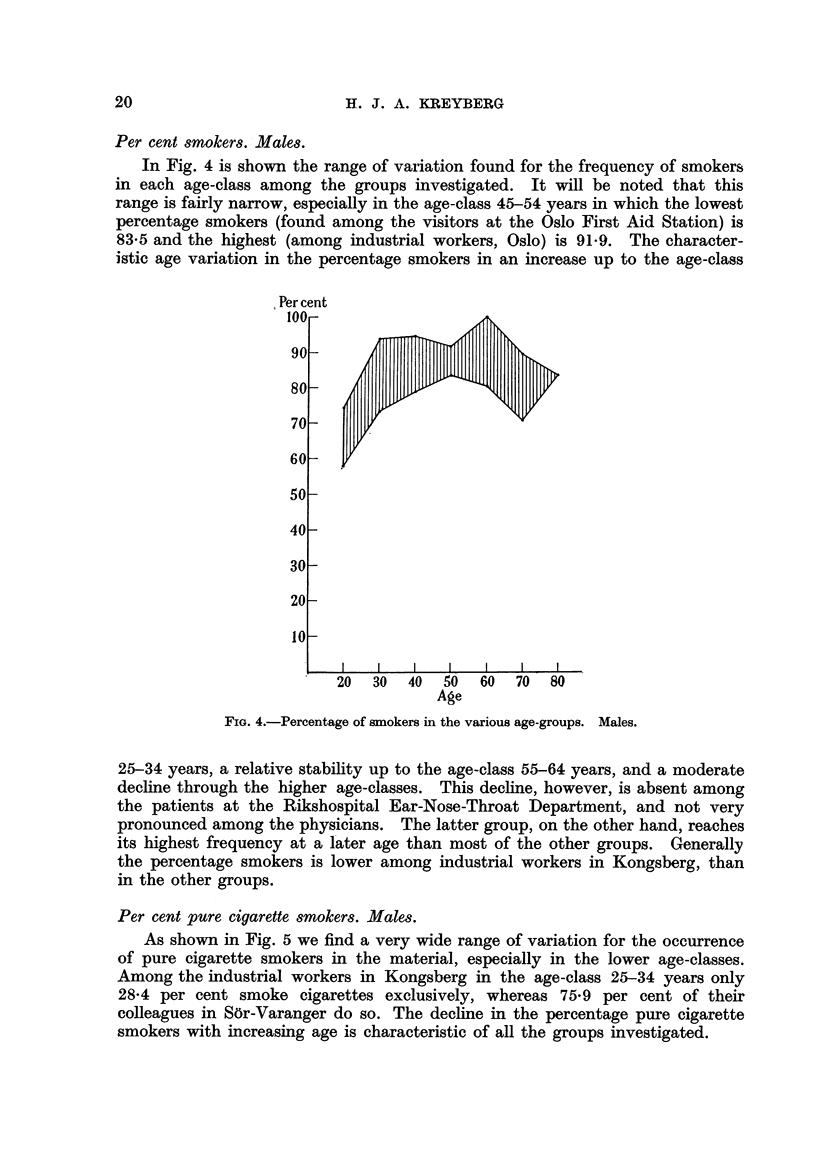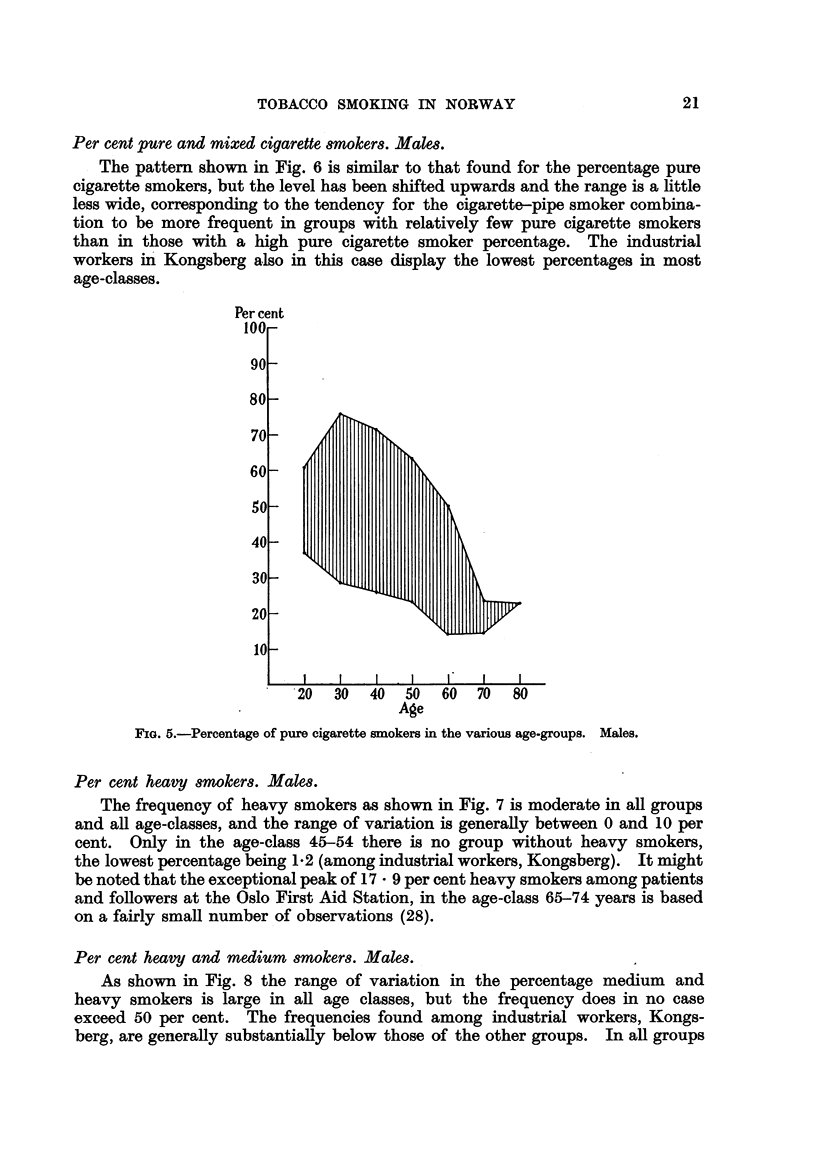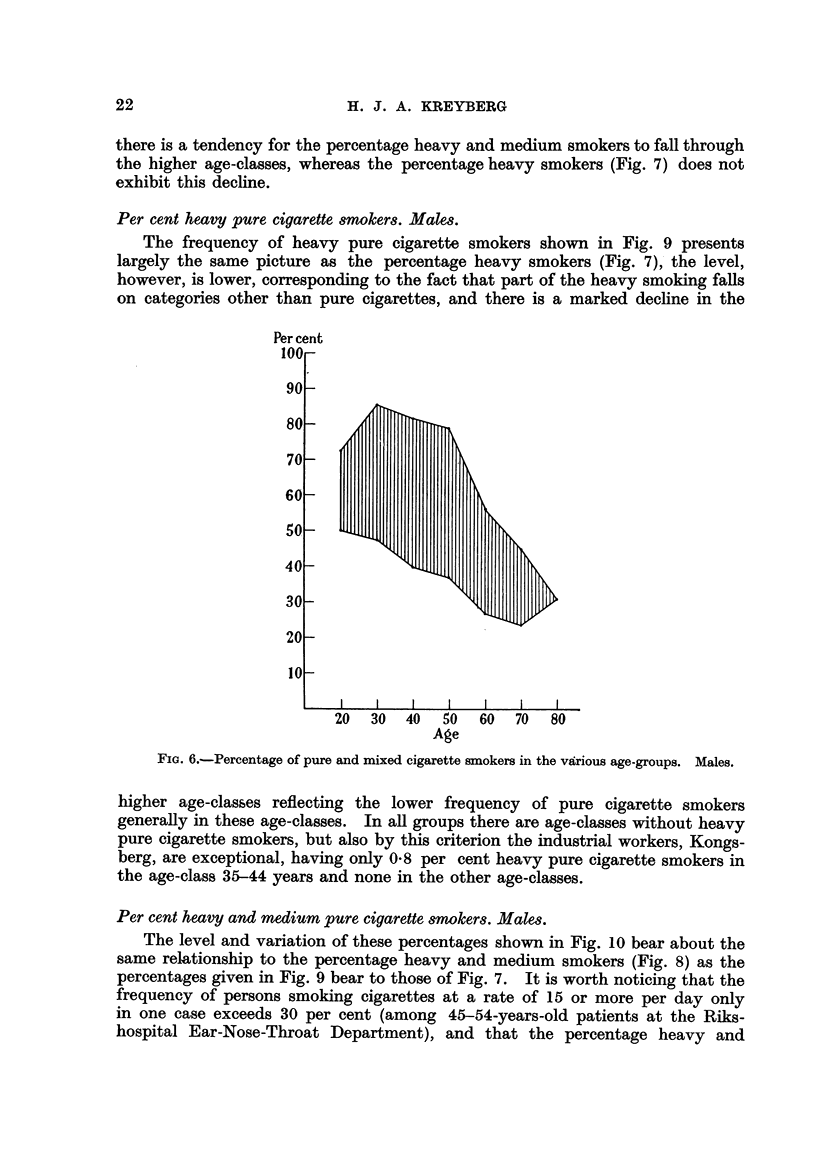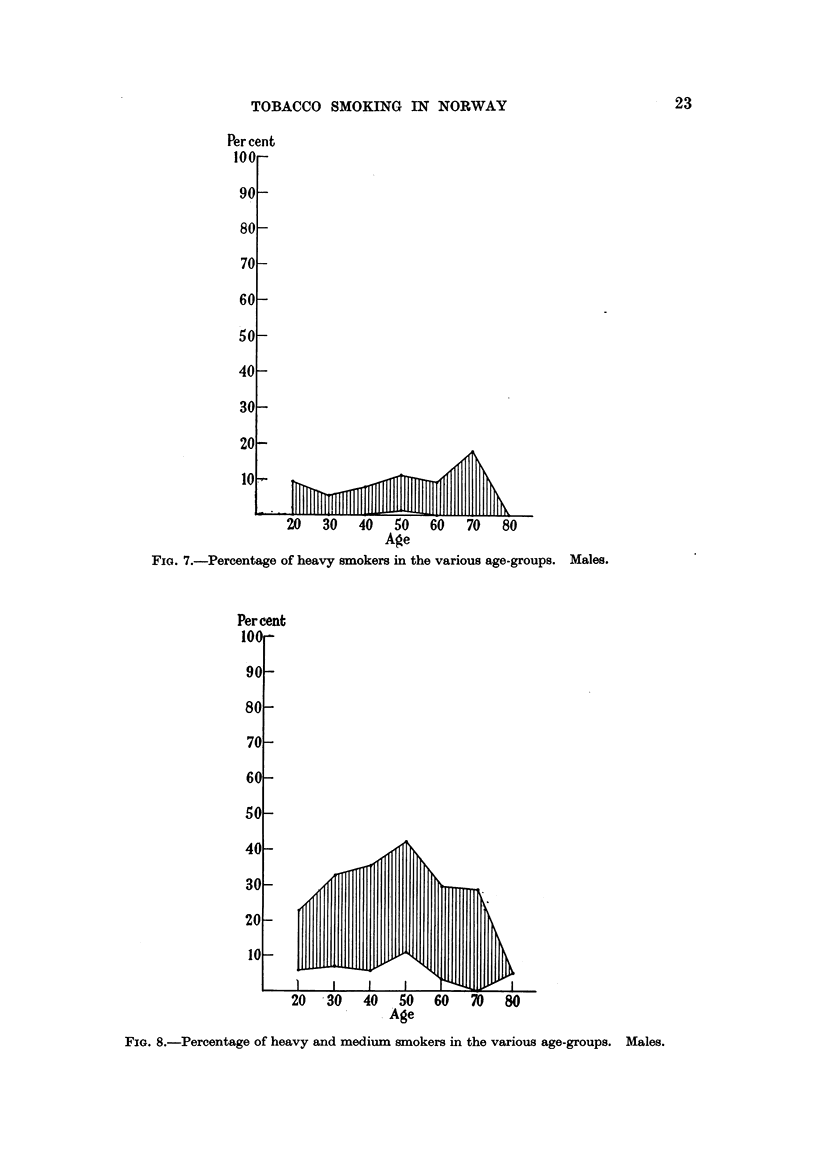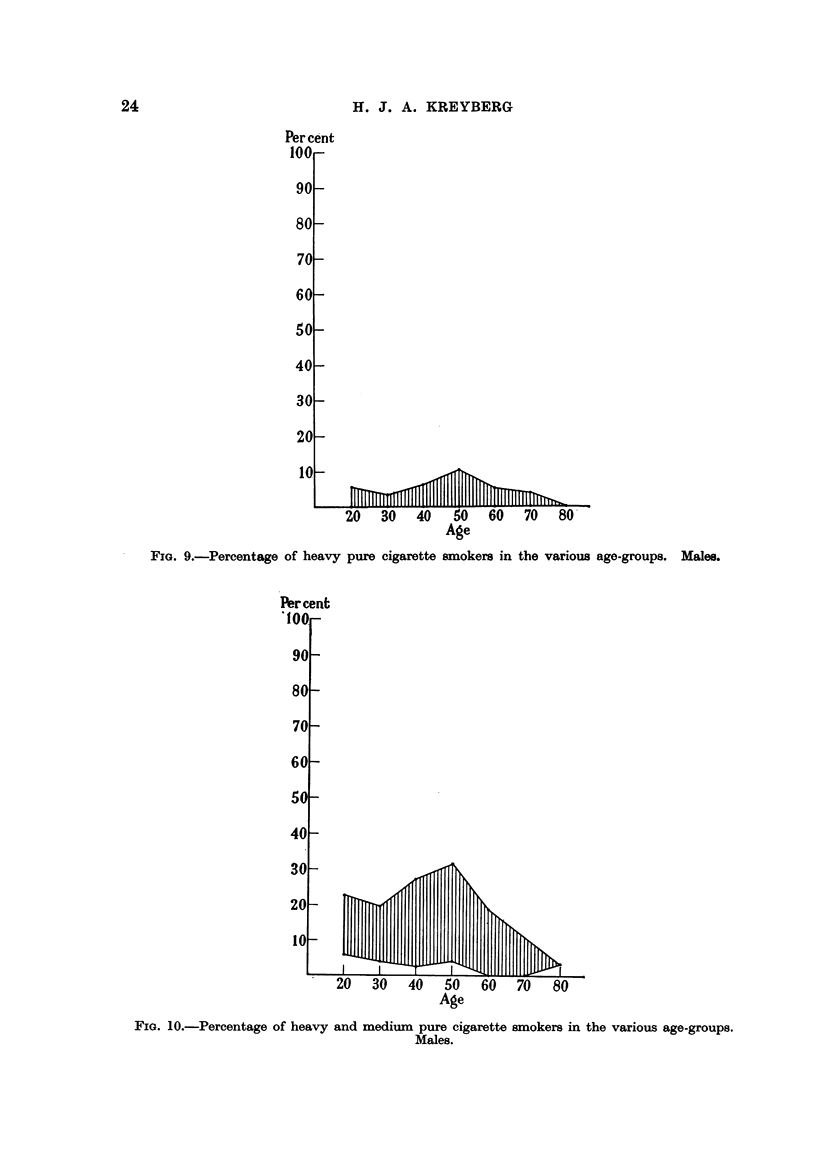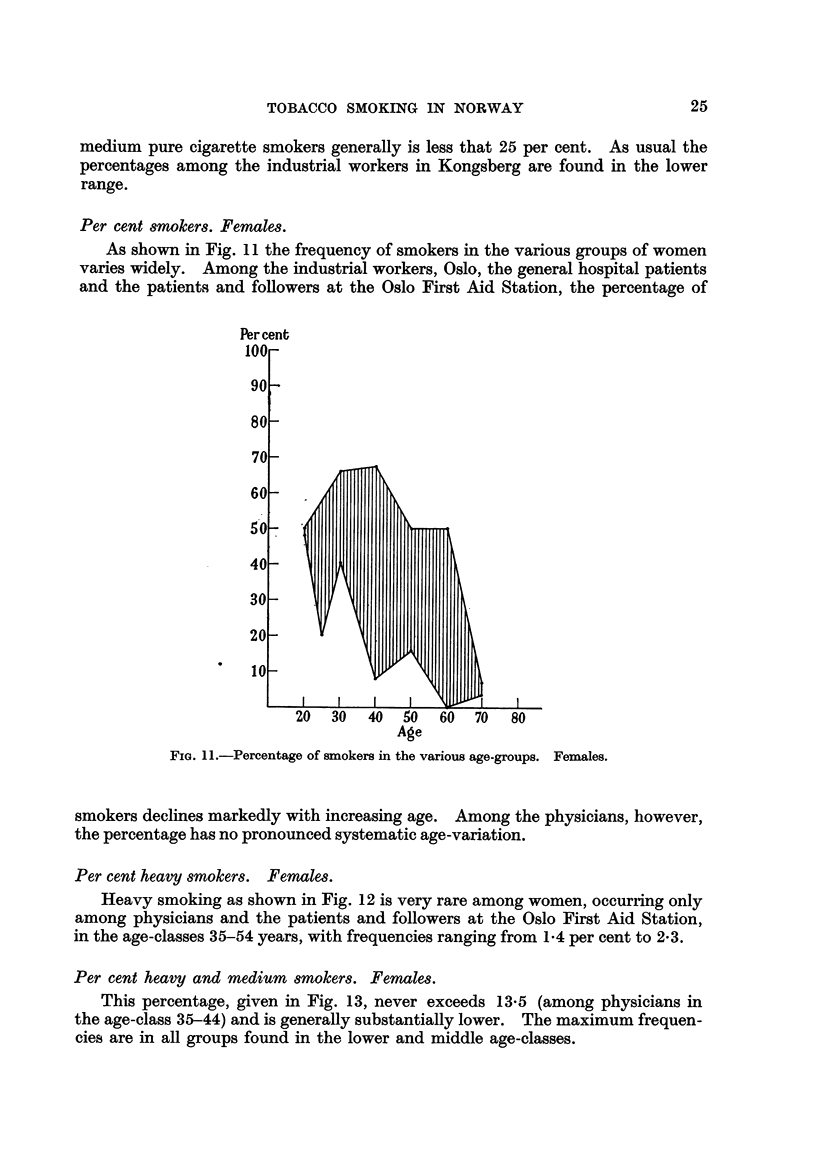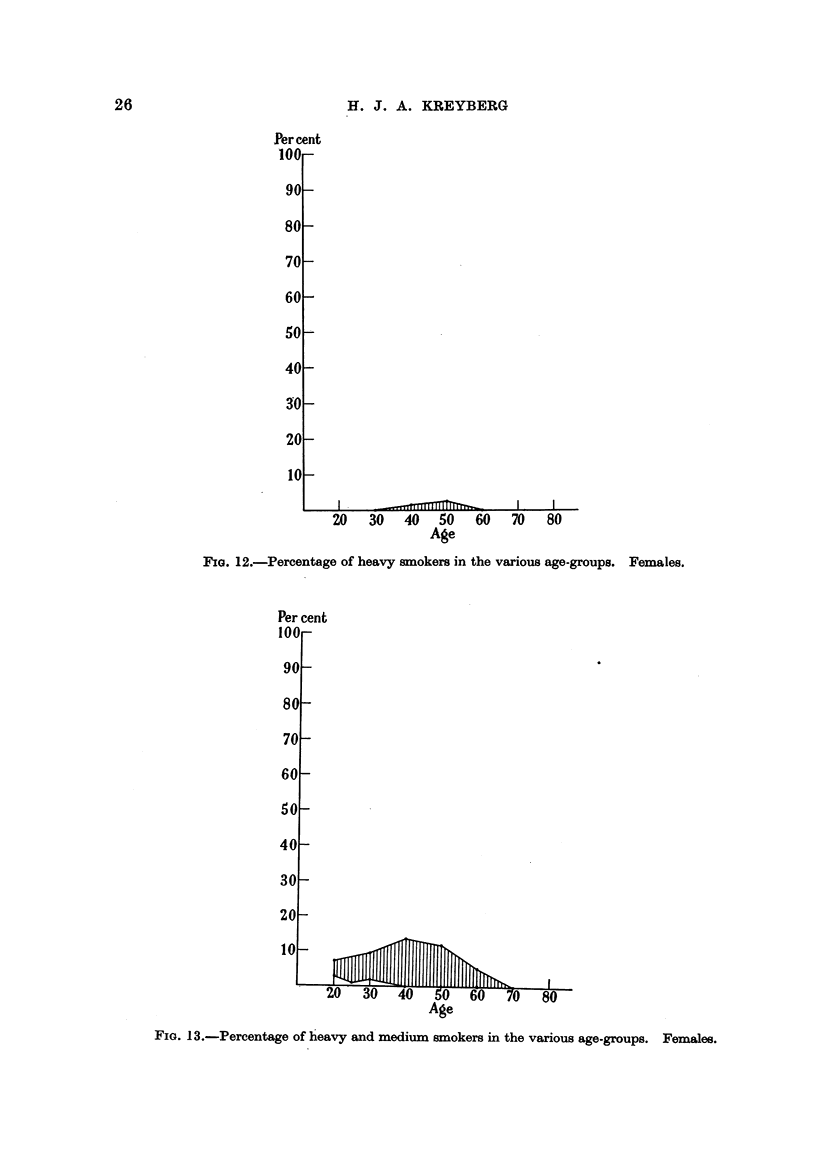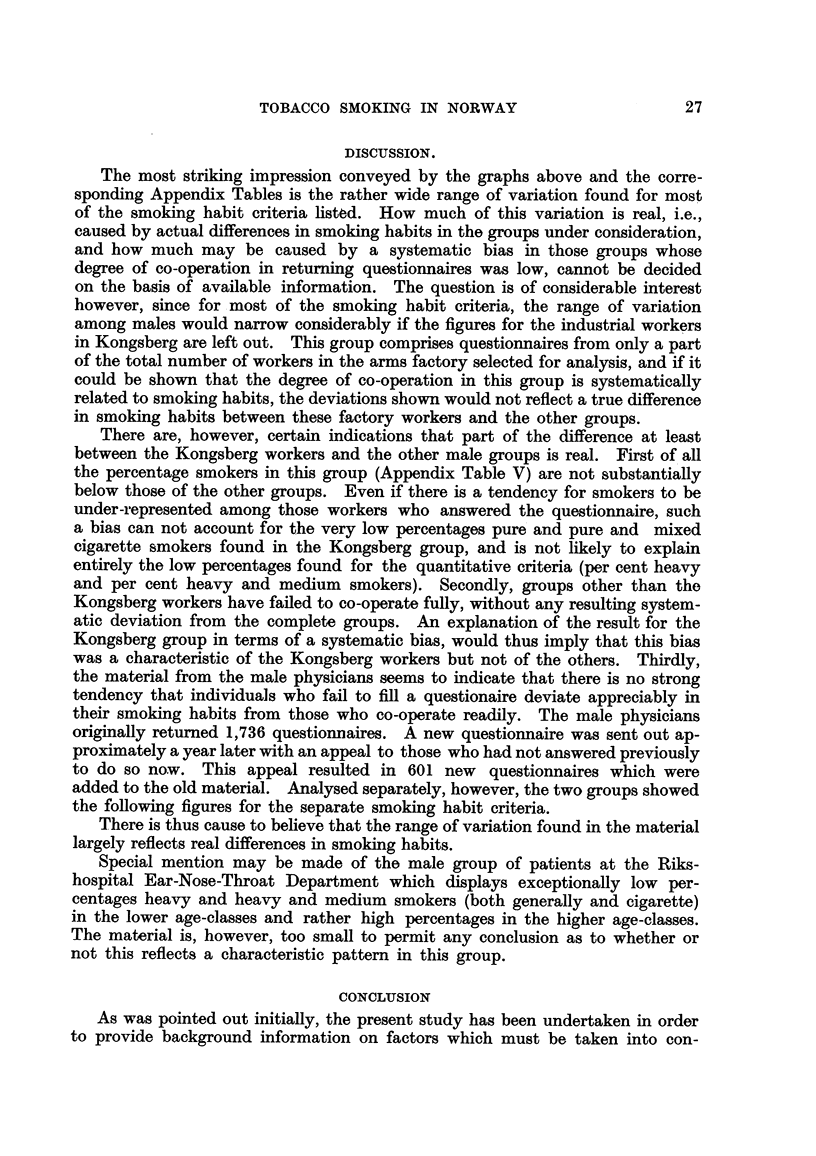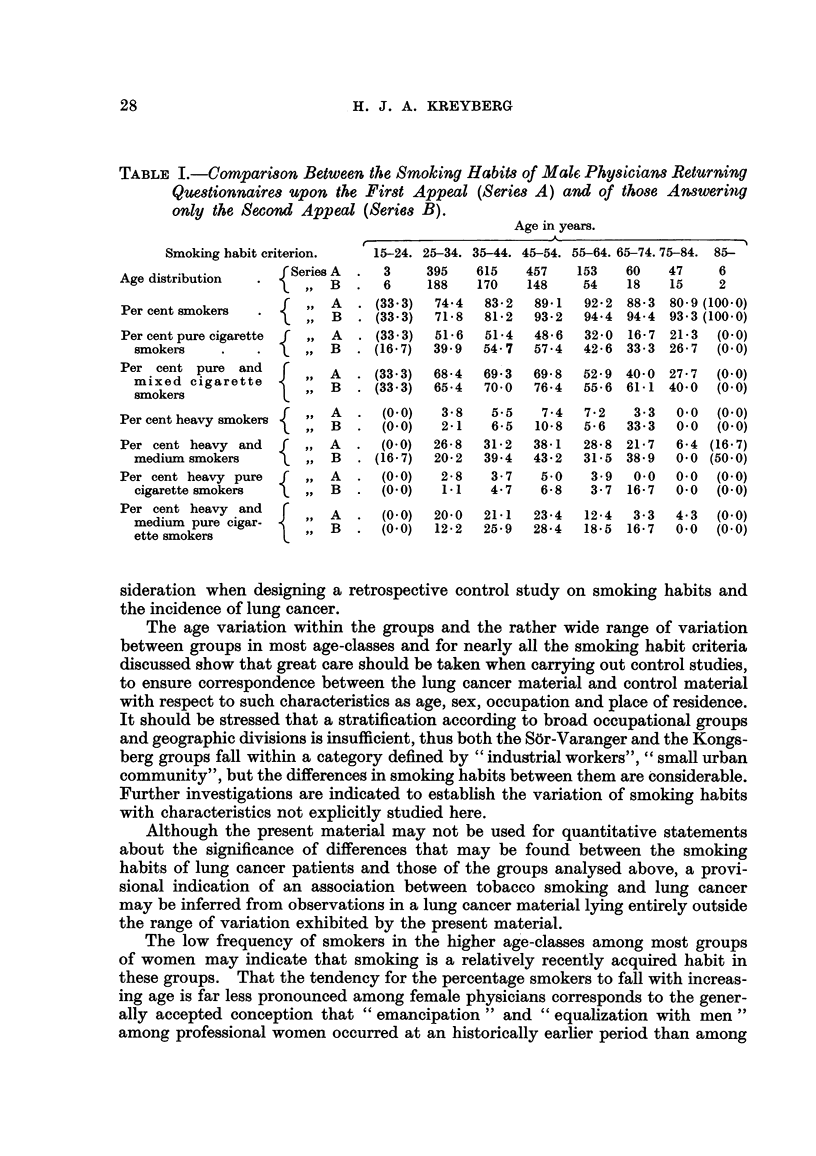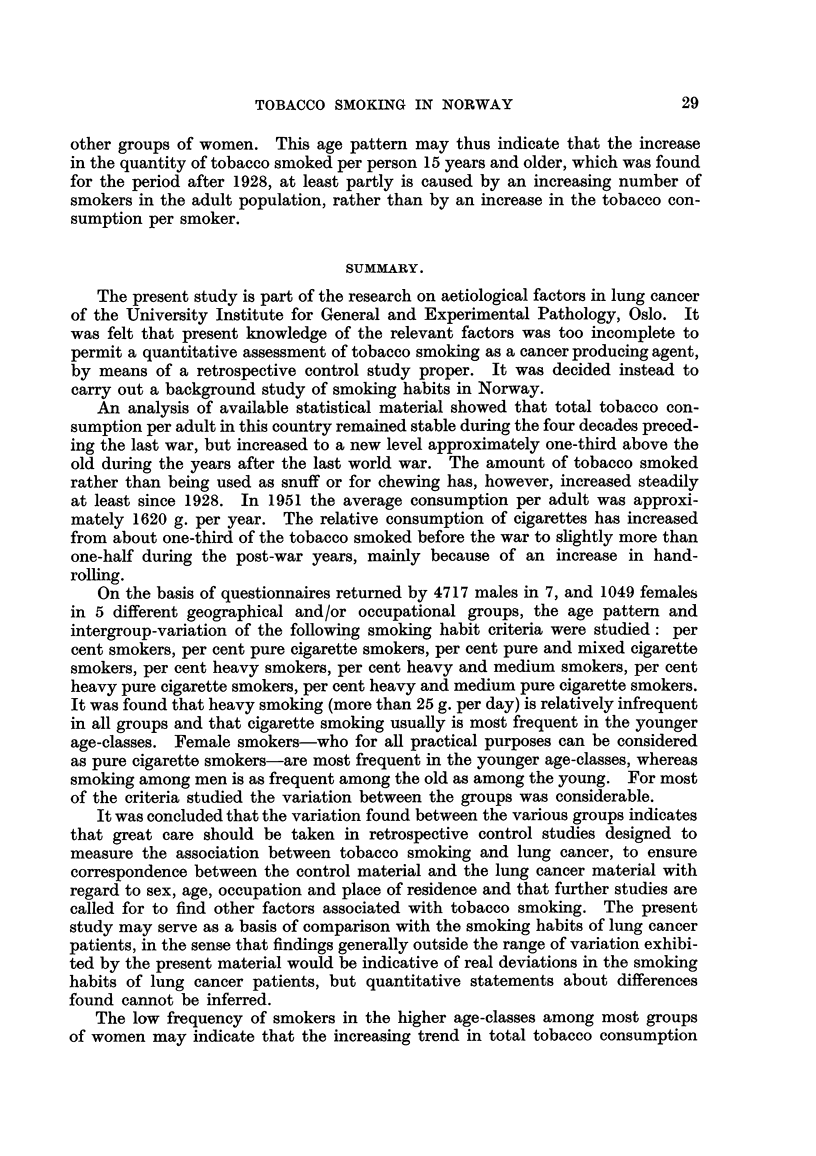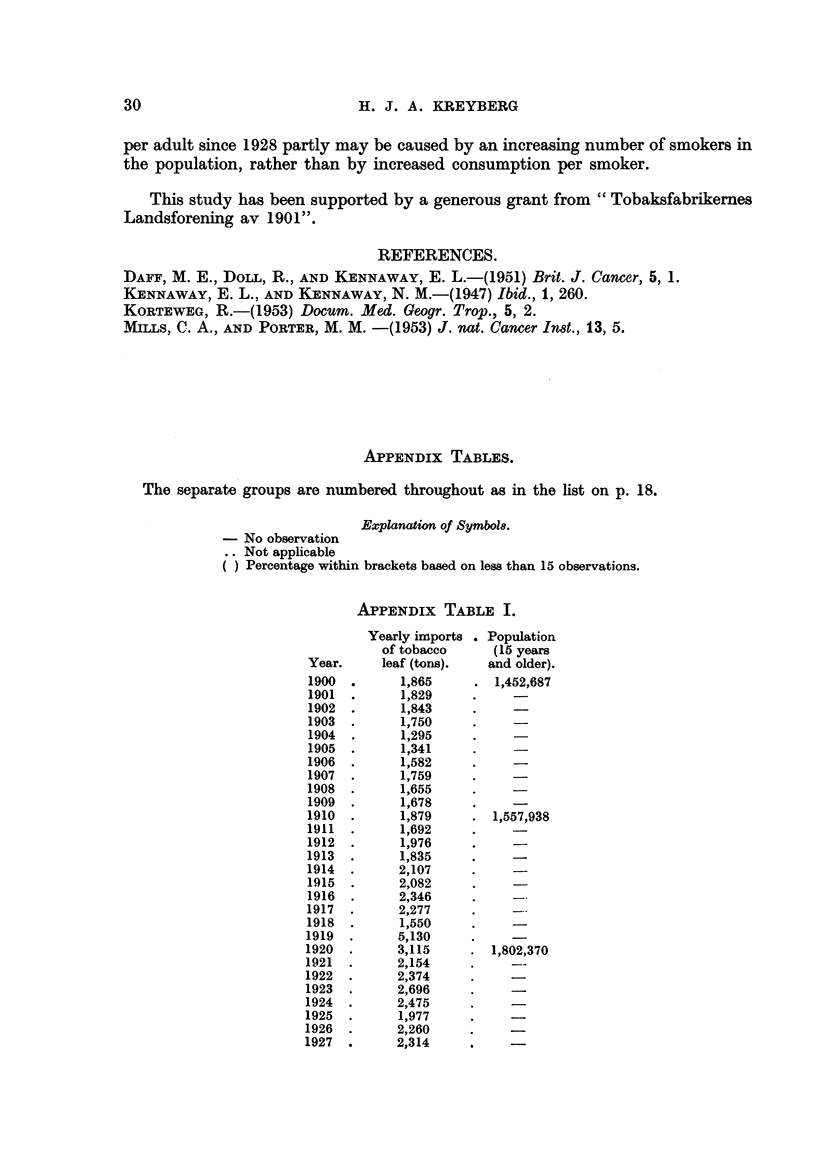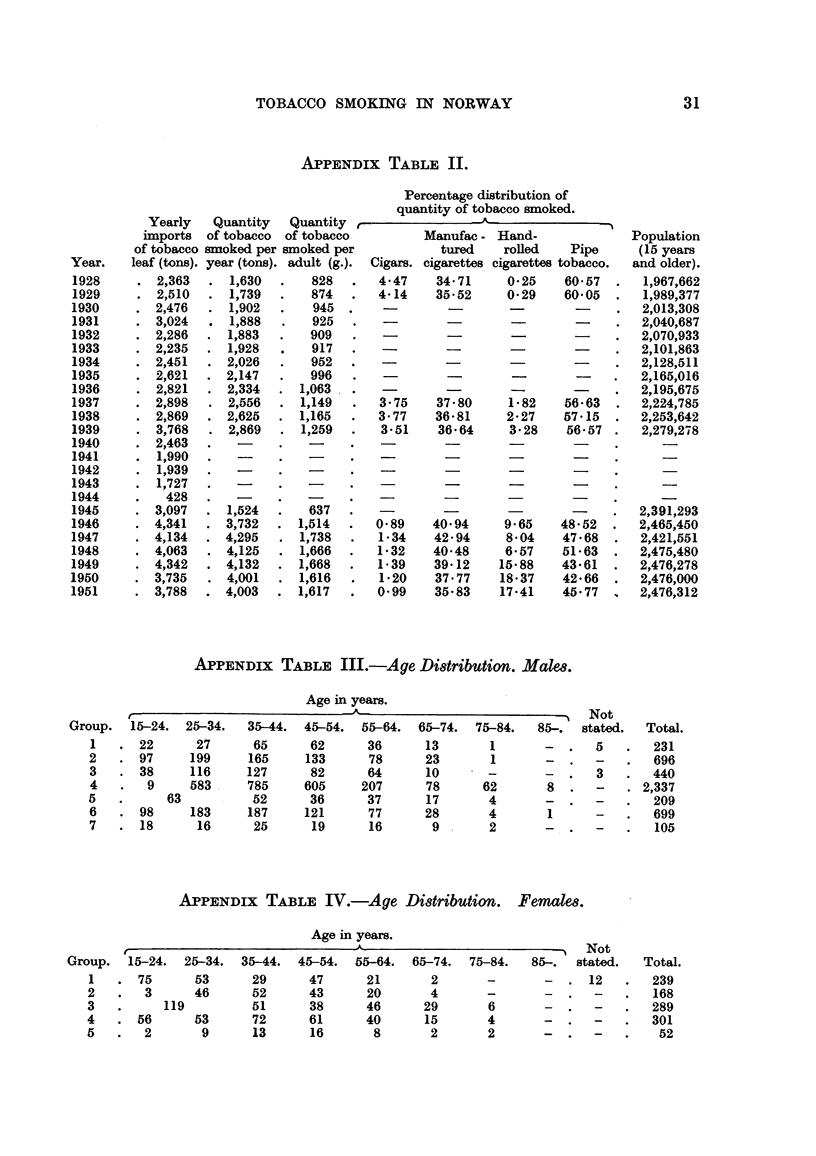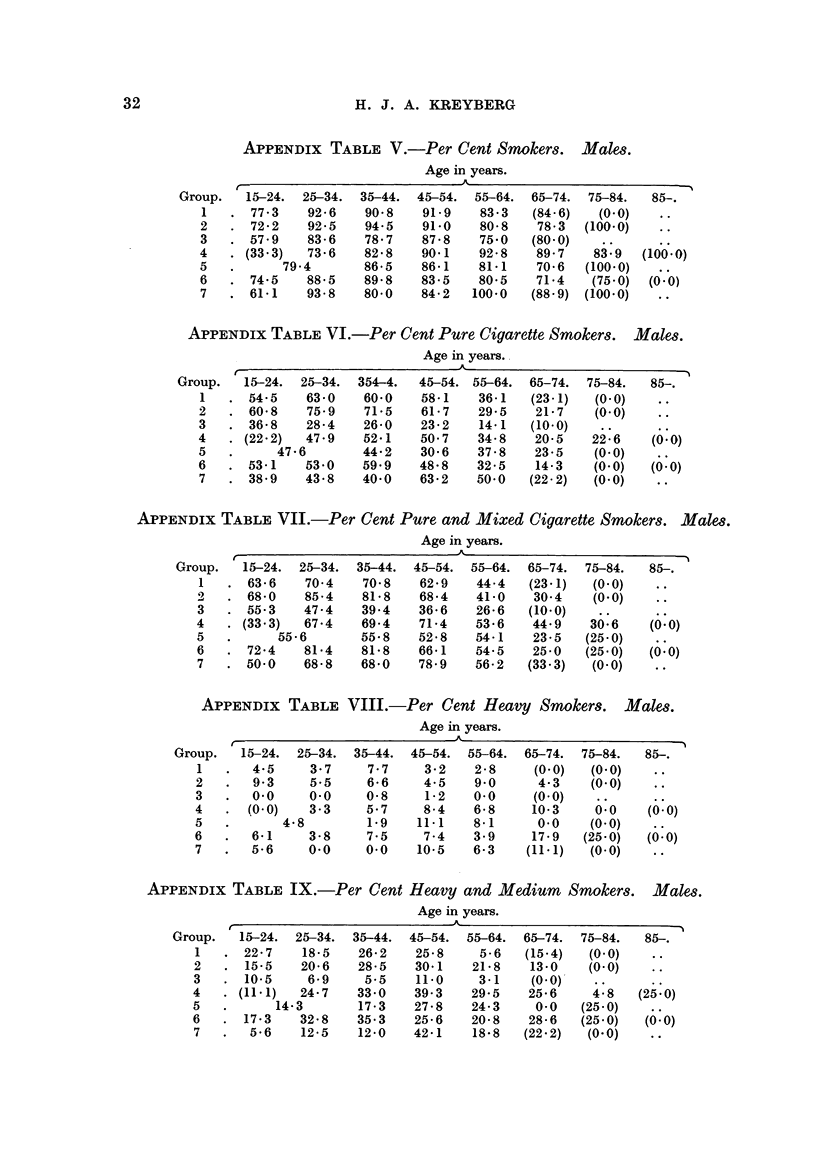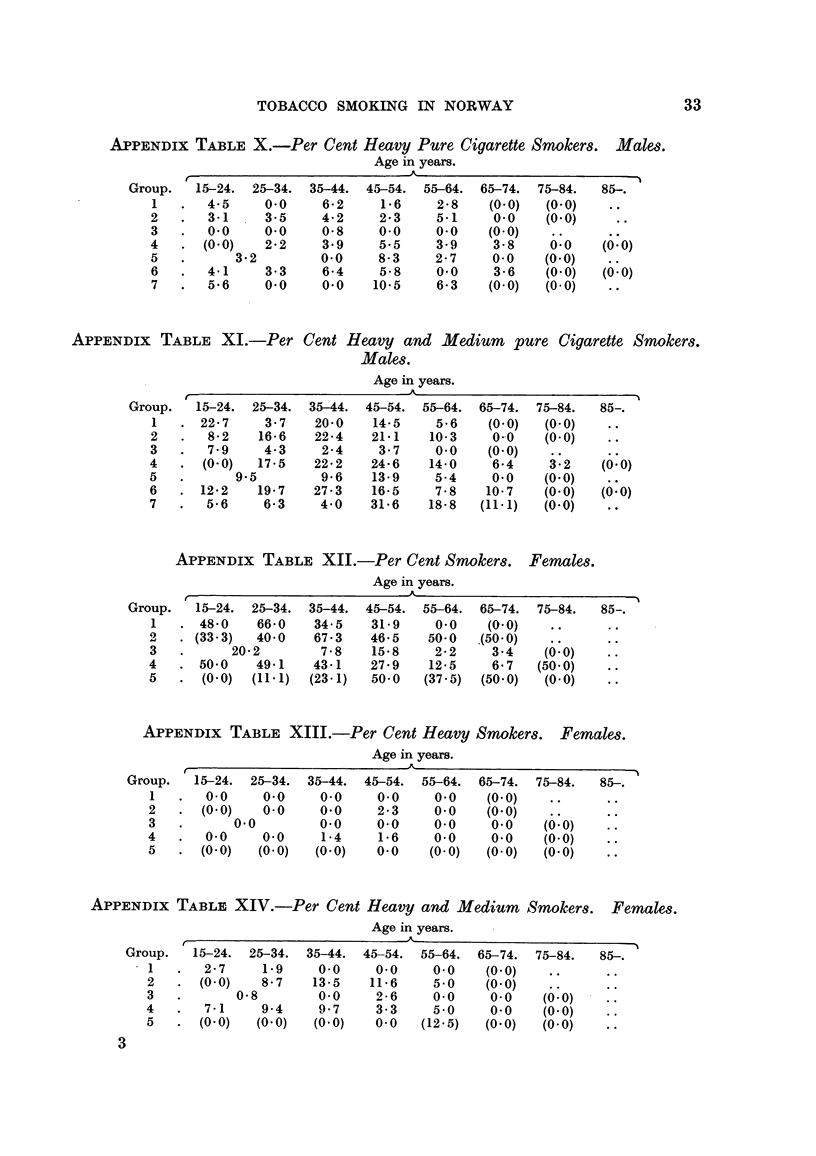# A Study of Tobacco Smoking in Norway

**DOI:** 10.1038/bjc.1954.2

**Published:** 1954-03

**Authors:** H. J. A. Kreyberg


### A STUDY OF TOBACCO SMOKING IN NORWAY.

H. J. A. KREYBERG.

*From the Institutt for Generell og Eksperimentell Patologi, Universitetet i Oslo.*

Received for publication December 28, 1953.

THE present paper is part of a research programme on aetiological factors in lung cancer, which is currently being carried out at the University Institute for General and Experimental Pathology in Oslo.

A study of the smoking habits of lung cancer patients for the purpose of ascertaining the rôle of tobacco as a causative factor poses the problem of obtaining an adequate control material. For a number of reasons discussed below it was found that to launch a control study proper at the present stage of the research would entail considerable practical difficulties and, above all, so many unknown factors, as to render its value as a basis for detailed statistical computation highly doubtful. It was decided, therefore, preliminarily to assemble available data on tobacco consumption in this country and to collect information on the smoking habits of geographically and occupationally distinct groups of the population as a pilot investigation for more solidly founded control studies which might be required at a later stage of the research programme, and as a provisional background for an evaluation of the smoking habits of lung cancer patients. It was felt that such a procedure, furthermore, would be likely to reveal any grossly excessive smoking that might be present among lung cancer patients, even if, at any specified level of probability, nothing definite could be said about the significance of differences found.

When the cancer producing effect of an environmental factor, such as tobacco smoking, is studied by comparing the smoking habits of cancer patients with those of a control material, the latter must satisfy specific requirements if valid quantitative conclusions are to be reached. In the strictest sense these requirements include the absence of lung cancer in the control material. However, so long as the incidence of lung cancer is small in all population groups (not selected according to whether lung cancer is present or not), the presence of concealed and potential lung cancer in the control material, which hardly can be avoided in practical applications, will not influence the results materially, and may probably be disregarded. This problem will not be dwelt upon in the present discussion ; individuals without diagnosed lung cancer will, in fact, be considered as non lung cancer cases.

More formidable difficulties are encountered in satisfying the following criteria : (*a*) the control material should correspond to the cancer patient group with respect to all factors associated both with the incidence of lung cancer and with the level of smoking, (*b*) the control material should not be selected according to any *other* factor, unrelated to the incidence of cancer, absent in the cancer patient group, which is associated with the level of smoking.

These requirements are not likely to be satisfied in any actual control study for the reason that the relevant factors largely are unknown. Even if correspondence is established between the patient group and the control material with regard to such factors as age and sex composition, distribution of broad occupational groups and place of residence, which may influence smoking habits and/or lung cancer frequency, and a large number of factors clearly may be considered irrelevant, a field of uncertainty of considerable extent still remains.

Two points of practical importance emerge from the above discussion. The first is that control material consisting of non lung cancer cases among patients in a chest department, or even among hospital patients generally, may introduce a bias if, for instance, the factor " visiting a chest department for reasons other than lung cancer "—present in a control material but not among the lung cancer patients—is associated with the level of tobacco smoking. There may thus be a tendency towards excessive smoking in such a control material, or patients in chest departments may on the contrary tend to be persons whose reaction even to mild forms of chest trouble is a visit to the hospital, and who are careful not to expose themselves to irritants such as tobacco. If both these tendencies are present among the patients, they may neutralize each other, but the net result will obviously depend on their relative occurrence in the control material. Similar objections may be brought against the use of hospital patients generally as control material. This point has been stressed by Mills and Porter (1953) who believe that the bias probably will obscure an excess of smoking among lung cancer cases. At least, it is not established that hospital patients do not differ systematically from the general population with regard to smoking habits.

The second point to be made is that a careful survey of all possible cancer producing factors must precede the instigation of a control study, and that preliminary surveys are needed to establish the relationships between these factors and the level of tobacco smoking.

There is thus *a priori* reason to consider the need for relevant definitions and differentiation of occupational groups among the cancer patients as well as in the control material, in view of the frequently indicated possibilities of specific occupational hazards with respect to lung cancer. Assume, for instance, that a particular type of industrial occupation has a definite but unrecognized excess incidence of lung cancer, and that the carcinogenic agent is connected with the nature of the work. Assume further that this particular occupation also is associated with an excess of tabocco smoking, because of higher pay, better opportunities of smoking during working hours, psychological stress or other causes. If the occupational selection criteria are such that this occupation does not explicitly constitute a stratum in the control material it may be under-represented in a control material with random selection within each stratum, and the controls may, for the separate occupational groups and *in toto*, show a lower smoking level than the cancer patient group. Such a difference will clearly be misleading for an evaluation of the carcinogenic effect of tobacco smoking. If, on the other hand, the particular occupation in our example is associated with an exceptionally low smoking level, a possibly significant excess smoking in the cancer patient group may be obscured. An example of such possibilities has been demonstrated, among others by Kennaway and Kennaway (1947), as regards lung carcinoma in coal miners.

It would appear that extensive research is called for to establish relevant definitions of occupational groups and the relationship between tobacco smoking and possible cancer producing types of occupation.

*Trends in Norwegian Tobacco Consumption.*

An indicator of tobacco consumption going back to the last century are the yearly imports of raw tobacco. These import quantities, which fairly accurately correspond to the amount of tobacco actually used in internal production, have a remarkably constant trend during the period from 1901 to 1940 when calculated per person 15 years and older in the population. After the last world war the level is about one-third higher. In Fig. 1 the solid line represent imports of raw

FIG. 1.—Imports of tobacco leaf and quantity of tobacco smoked.

——————— Yearly imports of tobacco leaf in tons.
— — — — — Population, 15 years and older.
. . . . . . . Tobacco smoked per year in tons.

tobacco in tons per year, and the heavy dotted line the population 15 years and older. The scales are adjusted so that constant imports at the 1901-level, per persons 15 years and older, would have coincided with the population curve. In view of the fact that net imports of finished tobacco products have been of negligible importance since the 1920's and that they, at the beginning of the period under consideration, played a greater rôle, it may be concluded that the total tobacco consumption per person 15 years and older, during the period after 1900, has not shown any sharp or substantial increases before the recent post-war years. This stability even seems to extend as far back as about 1865.

This, however, does not permit the conclusion that the quantity of tobacco actually smoked has been equally constant. Relatively complete data on the composition of tobacco consumption are available only after 1927. Since then, tobacco smoked, i.e., cigars, cigarettes and pipe tobacco, has accounted for a steadily increasing proportion of the total tobacco consumption at the expense of chewing tobacco and snuff, as shown in Fig. 1, where the dotted line represents the amounts smoked, in tons per year. The amount smoked is here calculated as production plus imports minus exports of cigars, cigarettes and pipe tobacco. Year to year inventory changes have not been corrected for. These changes will be small, however, and without influence on the trend, even if the yearly figures may contain erratic deviations from the true consumption level. It should be permissible to assume that chewing tobacco and snuff played a greater rôle before 1927. Even if some of the chewing tobacco in fact has been smoked in pipe, it is reasonable to assume that the amount of tobacco smoked, per person 15 years and older, has increased to some extent during the whole period from 1901 to 1940.

Fig. 2.—Consumption of smoking tobacco in grams per person 15 years and older.

After the last world war the yearly amount of tobacco smoked, i.e., cigars, cigarettes and pipe tobacco, has reached a level of approximately 1650 g. per person 15 years and older, or some 40 per cent above the immediate pre-war level, as shown in Fig. 2. This is considerably less than the *per capita* figures for tobacco consumption, for instance in Great Britain or Switzerland, quoted by Daff, Doll and Kennaway (1951) and for the Netherlands quoted by Korteweg (1953). Since 1947 there has been a slightly decreasing tendency.

The distribution of the tobacco smoked on the three types : cigars, cigarettes and pipe tobacco is known only for the years after 1927. The percentage distribution by weight is shown in Fig. 3 for 1928–29, 1937–39 and the post-war years. The black parts of the columns represent the proportion of ready made cigarettes, which was 34·7 per cent in 1928, rose to 42·9 per cent in 1947 and has since declined to 35·8 per cent in 1951. On the basis of sales tax returns for cigarette paper sold, it is possible to estimate the consumption of hand-rolled cigarettes

(the heavily shaded parts of the columns). Altogether total cigarette consumption, which accounted for 35·0 per cent of the total smoked in 1928 rose to 56·1 per cent in 1950 and has since fallen slightly to 53·2 per cent in 1951. Although the estimates concerning hand-rolled cigarettes are uncertain, there has doubtless been an increase in the relative share of cigarettes in tobacco smoked, particularly after the war, and this increase is almost wholly due to the greater prevalence of hand-rolling. It is reasonable to assume that the share of cigarettes increased

FIG. 3.—Composition of smoking tobacco consumption in Norway.

Cigars.

Pipe

Hand-rolled cigarettes

Manufactured cigarettes.

even between1901 and 1927 (for instance in 1909 cigarettes accounted for approximately 10 per cent of the internal tobacco production), but the information is too incomplete to establish the pattern of growth during this period.

The consumption share of cigars, represented by the vertically shaded top part of the column, the rest being pipe tobacco consumption, has been small after 1927 and has declined from 4·5 per cent in 1928 to 1·0 per cent in 1951. Just after the turn of the century, however, cigars seem to have played a greater rôle ; in 1909, for instance, cigars accounted for some 15-20 per cent of the internal production.

Whereas total tobacco consumption per person 15 years and older thus generally remained stable between 1901 and 1940, there was an increase in the amount smoked. Since the last world war smoking has reached a new and higher level, with a marked increase in the relative share of cigarettes, mainly caused by an increase in hand-rolling.

### *Smoking Habits in Certain Population Groups.*

The material analysed consists of questionnaires on smoking habits returned from a number of geographically and occupationally distinct groups in the population. The questionnaire has been practically identical with that used by Kennaway and Doll. The total material is distributed as follows among the separate groups. (See also tables in the Appendix.)

*Males.*

| Group. | No. of questionnaires. |
|---|---|
| 1. Industrial workers, Oslo (shipbuilding) | 231 |
| 2. Industrial workers, Sör-Varanger (several trades) | 696 |
| 3. Industrial workers, Kongsberg (arms factory) | 440 |
| 4. Physicians, whole country | 2337 |
| 5. General Hospital patients, mainly rural | 209 |
| 6. Patients and followers, Oslo City First Aid Station | 699 |
| 7. Patients, Ear-Nose-Throat Department, the Rikshospital (whole country) | 105 |
| Total | 4717 |

*Females.*

| Group | No. of questionnaires. |
|---|---|
| 1. Industrial workers, Oslo (chocolate factory) | 239 |
| 2. Physicians, whole country | 168 |
| 3. General hospital patients, mainly rural | 289 |
| 4. Patients and followers, Oslo City First Aid Station | 301 |
| 5. Patients, Ear-Nose-Throat Department, the Rikshospital (whole country) | 52 |
| Total | 1049 |

*Kongsberg* is a small industrial town in central southern Norway, *Sör-Varanger* (Kirkenes) a small industrial and mining community (iron-ore) in the extreme north.

In the Groups 1, 3, 4 and 5 among the males and 1, 2 and 3 among the females the number of returned questionnaires falls substantially short of the total number of individuals in the groups selected. The questionnaires were filled in by the members of these groups themselves and the degree of co-operation varied considerably. However, in the case of the patients and followers (friends or relatives who accompanied them) at the Oslo First Aid Station and the patients at the Ear-Nose-Throat Department at the Rikshospital all questionnaires were filled in by one interviewer who approached all patients and/or followers reporting at these two institutions during the periods when he was present (in July 1953). The number of refusals was negligible in both cases, and the possibility of a bias in these groups caused by systematic differences in the smoking habits between persons willing to give information and those unwilling to do so, can be excluded.

This is also the case in Group 2 among the males, in which the coverage was practically complete. On the other hand an unknown bias may be present in the other groups, caused by varying degrees of co-operation.

*Definitions.*

Each of the above-mentioned groups were divided into age-classes with a class interval of 10 years. The age distribution of the separate groups is given in Appendix Tables III and IV. For each group and age-class the percentage incidence of each of the following were computed.

For males :

1. Smokers.
2. Pure cigarette smokers.
3. Pure and mixed cigarette smokers.
4. Heavy smokers.
5. Heavy and medium smokers.
6. Heavy pure cigarette smokers.
7. Heavy and medium pure cigarette smokers.

For females :

1. Smokers.
2. Heavy smokers.
3. Heavy and medium smokers.

The term smoker designates a person who has smoked as much as 1 g. of tobacco (in any form) daily for at least 1 year. A pure cigarette smoker is a smoker whose consumption of tobacco smoked other than cigarettes, is insufficient alone to establish him as a smoker. A mixed cigarette smoker is any smoker who is not a pure cigarette smoker, but whose consumption of cigarettes alone is sufficient to establish him as a smoker. Heavy smokers are all smokers whose daily consumption is 25 g. or more. Heavy and medium smokers are all smokers whose daily consumption is 15 g. or more. One cigarette is conventionally set equal to 1 g. No differentiation has been made between hand-rolled and manufactured cigarettes. One cigar is counted as 5 g. The amount of pipe tobacco smoked is usually in the returned questionnaires stated as grams (or number of packages, each 50 g.) per week. In the case of females, smokers are synonymous with pure cigarette smokers throughout. There were only three cases altogether of women (all doctors), who had at some time or other smoked cigars and/or pipe in addition to cigarettes, and these have been counted as pure cigarette smokers.

The percentage distributions according to the criteria listed above are given in Appendix Tables V–XIV. In the following pages summary graphs and a brief commentary on the distribution according to the separate smoking habit criteria will be given. The shaded areas of the graphs indicate the range of variation found between the groups for each of the criteria listed. The upper boundary thus connects the maximum values found in each age-class and the lower, similarly, the minimum values. All percentages based on less than 15 observations, given in brackets in the Appendix Tables, have however, been excluded in the graphs.

*Per cent smokers. Males.*

In Fig. 4 is shown the range of variation found for the frequency of smokers in each age-class among the groups investigated. It will be noted that this range is fairly narrow, especially in the age-class 45–54 years in which the lowest percentage smokers (found among the visitors at the Oslo First Aid Station) is 83·5 and the highest (among industrial workers, Oslo) is 91·9. The characteristic age variation in the percentage smokers in an increase up to the age-class 25–34 years, a relative stability up to the age-class 55–64 years, and a moderate decline through the higher age-classes. This decline, however, is absent among the patients at the Rikshospital Ear-Nose-Throat Department, and not very pronounced among the physicians. The latter group, on the other hand, reaches its highest frequency at a later age than most of the other groups. Generally the percentage smokers is lower among industrial workers in Kongsberg, than in the other groups.

Fig. 4.—Percentage of smokers in the various age-groups. Males.

*Per cent pure cigarette smokers. Males.*

As shown in Fig. 5 we find a very wide range of variation for the occurrence of pure cigarette smokers in the material, especially in the lower age-classes. Among the industrial workers in Kongsberg in the age-class 25–34 years only 28·4 per cent smoke cigarettes exclusively, whereas 75·9 per cent of their colleagues in Sör-Varanger do so. The decline in the percentage pure cigarette smokers with increasing age is characteristic of all the groups investigated.

*Per cent pure and mixed cigarette smokers. Males.*

The pattern shown in Fig. 6 is similar to that found for the percentage pure cigarette smokers, but the level has been shifted upwards and the range is a little less wide, corresponding to the tendency for the cigarette–pipe smoker combination to be more frequent in groups with relatively few pure cigarette smokers than in those with a high pure cigarette smoker percentage. The industrial workers in Kongsberg also in this case display the lowest percentages in most age-classes.

FIG. 5.—Percentage of pure cigarette smokers in the various age-groups. Males.

*Per cent heavy smokers. Males.*

The frequency of heavy smokers as shown in Fig. 7 is moderate in all groups and all age-classes, and the range of variation is generally between 0 and 10 per cent. Only in the age-class 45–54 there is no group without heavy smokers, the lowest percentage being 1·2 (among industrial workers, Kongsberg). It might be noted that the exceptional peak of 17 · 9 per cent heavy smokers among patients and followers at the Oslo First Aid Station, in the age-class 65–74 years is based on a fairly small number of observations (28).

*Per cent heavy and medium smokers. Males.*

As shown in Fig. 8 the range of variation in the percentage medium and heavy smokers is large in all age classes, but the frequency does in no case exceed 50 per cent. The frequencies found among industrial workers, Kongsberg, are generally substantially below those of the other groups. In all groups there is a tendency for the percentage heavy and medium smokers to fall through the higher age-classes, whereas the percentage heavy smokers (Fig. 7) does not exhibit this decline.

*Per cent heavy pure cigarette smokers. Males.*

The frequency of heavy pure cigarette smokers shown in Fig. 9 presents largely the same picture as the percentage heavy smokers (Fig. 7), the level, however, is lower, corresponding to the fact that part of the heavy smoking falls on categories other than pure cigarettes, and there is a marked decline in the

Fig. 6.—Percentage of pure and mixed cigarette smokers in the various age-groups. Males.

higher age-classes reflecting the lower frequency of pure cigarette smokers generally in these age-classes. In all groups there are age-classes without heavy pure cigarette smokers, but also by this criterion the industrial workers, Kongsberg, are exceptional, having only 0·8 per cent heavy pure cigarette smokers in the age-class 35–44 years and none in the other age-classes.

*Per cent heavy and medium pure cigarette smokers. Males.*

The level and variation of these percentages shown in Fig. 10 bear about the same relationship to the percentage heavy and medium smokers (Fig. 8) as the percentages given in Fig. 9 bear to those of Fig. 7. It is worth noticing that the frequency of persons smoking cigarettes at a rate of 15 or more per day only in one case exceeds 30 per cent (among 45–54-years-old patients at the Rikshospital Ear-Nose-Throat Department), and that the percentage heavy and

FIG. 7.—Percentage of heavy smokers in the various age-groups. Males.

FIG. 8.—Percentage of heavy and medium smokers in the various age-groups. Males.

FIG. 9.—Percentage of heavy pure cigarette smokers in the various age-groups. Males.

FIG. 10.—Percentage of heavy and medium pure cigarette smokers in the various age-groups. Males.

medium pure cigarette smokers generally is less that 25 per cent. As usual the percentages among the industrial workers in Kongsberg are found in the lower range.

*Per cent smokers. Females.*

As shown in Fig. 11 the frequency of smokers in the various groups of women varies widely. Among the industrial workers, Oslo, the general hospital patients and the patients and followers at the Oslo First Aid Station, the percentage of

FIG. 11.—Percentage of smokers in the various age-groups. Females.

smokers declines markedly with increasing age. Among the physicians, however, the percentage has no pronounced systematic age-variation.

*Per cent heavy smokers. Females.*

Heavy smoking as shown in Fig. 12 is very rare among women, occurring only among physicians and the patients and followers at the Oslo First Aid Station, in the age-classes 35–54 years, with frequencies ranging from 1·4 per cent to 2·3.

*Per cent heavy and medium smokers. Females.*

This percentage, given in Fig. 13, never exceeds 13·5 (among physicians in the age-class 35–44) and is generally substantially lower. The maximum frequencies are in all groups found in the lower and middle age-classes.

Fig. 12.—Percentage of heavy smokers in the various age-groups. Females.

Fig. 13.—Percentage of heavy and medium smokers in the various age-groups. Females.

### DISCUSSION.

The most striking impression conveyed by the graphs above and the corresponding Appendix Tables is the rather wide range of variation found for most of the smoking habit criteria listed. How much of this variation is real, i.e., caused by actual differences in smoking habits in the groups under consideration, and how much may be caused by a systematic bias in those groups whose degree of co-operation in returning questionnaires was low, cannot be decided on the basis of available information. The question is of considerable interest however, since for most of the smoking habit criteria, the range of variation among males would narrow considerably if the figures for the industrial workers in Kongsberg are left out. This group comprises questionnaires from only a part of the total number of workers in the arms factory selected for analysis, and if it could be shown that the degree of co-operation in this group is systematically related to smoking habits, the deviations shown would not reflect a true difference in smoking habits between these factory workers and the other groups.

There are, however, certain indications that part of the difference at least between the Kongsberg workers and the other male groups is real. First of all the percentage smokers in this group (Appendix Table V) are not substantially below those of the other groups. Even if there is a tendency for smokers to be under-represented among those workers who answered the questionnaire, such a bias can not account for the very low percentages pure and pure and mixed cigarette smokers found in the Kongsberg group, and is not likely to explain entirely the low percentages found for the quantitative criteria (per cent heavy and per cent heavy and medium smokers). Secondly, groups other than the Kongsberg workers have failed to co-operate fully, without any resulting systematic deviation from the complete groups. An explanation of the result for the Kongsberg group in terms of a systematic bias, would thus imply that this bias was a characteristic of the Kongsberg workers but not of the others. Thirdly, the material from the male physicians seems to indicate that there is no strong tendency that individuals who fail to fill a questionaire deviate appreciably in their smoking habits from those who co-operate readily. The male physicians originally returned 1,736 questionnaires. A new questionnaire was sent out approximately a year later with an appeal to those who had not answered previously to do so now. This appeal resulted in 601 new questionnaires which were added to the old material. Analysed separately, however, the two groups showed the following figures for the separate smoking habit criteria.

There is thus cause to believe that the range of variation found in the material largely reflects real differences in smoking habits.

Special mention may be made of the male group of patients at the Rikshospital Ear-Nose-Throat Department which displays exceptionally low percentages heavy and heavy and medium smokers (both generally and cigarette) in the lower age-classes and rather high percentages in the higher age-classes. The material is, however, too small to permit any conclusion as to whether or not this reflects a characteristic pattern in this group.

### CONCLUSION

As was pointed out initially, the present study has been undertaken in order to provide background information on factors which must be taken into con-

TABLE I.—*Comparison Between the Smoking Habits of Male Physicians Returning Questionnaires upon the First Appeal (Series A) and of those Answering only the Second Appeal (Series B).*

| Smoking habit criterion. | | Age in years. 15–24. | 25–34. | 35–44. | 45–54. | 55–64. | 65–74. | 75–84. | 85– |
|---|---|---|---|---|---|---|---|---|---|
| Age distribution | Series A | 3 | 395 | 615 | 457 | 153 | 60 | 47 | 6 |
| | „ B | 6 | 188 | 170 | 148 | 54 | 18 | 15 | 2 |
| Per cent smokers | „ A | (33·3) | 74·4 | 83·2 | 89·1 | 92·2 | 88·3 | 80·9 | (100·0) |
| | „ B | (33·3) | 71·8 | 81·2 | 93·2 | 94·4 | 94·4 | 93·3 | (100·0) |
| Per cent pure cigarette smokers | „ A | (33·3) | 51·6 | 51·4 | 48·6 | 32·0 | 16·7 | 21·3 | (0·0) |
| | „ B | (16·7) | 39·9 | 54·7 | 57·4 | 42·6 | 33·3 | 26·7 | (0·0) |
| Per cent pure and mixed cigarette smokers | „ A | (33·3) | 68·4 | 69·3 | 69·8 | 52·9 | 40·0 | 27·7 | (0·0) |
| | „ B | (33·3) | 65·4 | 70·0 | 76·4 | 55·6 | 61·1 | 40·0 | (0·0) |
| Per cent heavy smokers | „ A | (0·0) | 3·8 | 5·5 | 7·4 | 7·2 | 3·3 | 0·0 | (0·0) |
| | „ B | (0·0) | 2·1 | 6·5 | 10·8 | 5·6 | 33·3 | 0·0 | (0·0) |
| Per cent heavy and medium smokers | „ A | (0·0) | 26·8 | 31·2 | 38·1 | 28·8 | 21·7 | 6·4 | (16·7) |
| | „ B | (16·7) | 20·2 | 39·4 | 43·2 | 31·5 | 38·9 | 0·0 | (50·0) |
| Per cent heavy pure cigarette smokers | „ A | (0·0) | 2·8 | 3·7 | 5·0 | 3·9 | 0·0 | 0·0 | (0·0) |
| | „ B | (0·0) | 1·1 | 4·7 | 6·8 | 3·7 | 16·7 | 0·0 | (0·0) |
| Per cent heavy and medium pure cigarette smokers | „ A | (0·0) | 20·0 | 21·1 | 23·4 | 12·4 | 3·3 | 4·3 | (0·0) |
| | „ B | (0·0) | 12·2 | 25·9 | 28·4 | 18·5 | 16·7 | 0·0 | (0·0) |

sideration when designing a retrospective control study on smoking habits and the incidence of lung cancer.

The age variation within the groups and the rather wide range of variation between groups in most age-classes and for nearly all the smoking habit criteria discussed show that great care should be taken when carrying out control studies, to ensure correspondence between the lung cancer material and control material with respect to such characteristics as age, sex, occupation and place of residence. It should be stressed that a stratification according to broad occupational groups and geographic divisions is insufficient, thus both the Sör-Varanger and the Kongsberg groups fall within a category defined by "industrial workers", "small urban community", but the differences in smoking habits between them are considerable. Further investigations are indicated to establish the variation of smoking habits with characteristics not explicitly studied here.

Although the present material may not be used for quantitative statements about the significance of differences that may be found between the smoking habits of lung cancer patients and those of the groups analysed above, a provisional indication of an association between tobacco smoking and lung cancer may be inferred from observations in a lung cancer material lying entirely outside the range of variation exhibited by the present material.

The low frequency of smokers in the higher age-classes among most groups of women may indicate that smoking is a relatively recently acquired habit in these groups. That the tendency for the percentage smokers to fall with increasing age is far less pronounced among female physicians corresponds to the generally accepted conception that " emancipation " and " equalization with men " among professional women occurred at an historically earlier period than among other groups of women. This age pattern may thus indicate that the increase in the quantity of tobacco smoked per person 15 years and older, which was found for the period after 1928, at least partly is caused by an increasing number of smokers in the adult population, rather than by an increase in the tobacco consumption per smoker.

### SUMMARY.

The present study is part of the research on aetiological factors in lung cancer of the University Institute for General and Experimental Pathology, Oslo. It was felt that present knowledge of the relevant factors was too incomplete to permit a quantitative assessment of tobacco smoking as a cancer producing agent, by means of a retrospective control study proper. It was decided instead to carry out a background study of smoking habits in Norway.

An analysis of available statistical material showed that total tobacco consumption per adult in this country remained stable during the four decades preceding the last war, but increased to a new level approximately one-third above the old during the years after the last world war. The amount of tobacco smoked rather than being used as snuff or for chewing has, however, increased steadily at least since 1928. In 1951 the average consumption per adult was approximately 1620 g. per year. The relative consumption of cigarettes has increased from about one-third of the tobacco smoked before the war to slightly more than one-half during the post-war years, mainly because of an increase in hand-rolling.

On the basis of questionnaires returned by 4717 males in 7, and 1049 females in 5 different geographical and/or occupational groups, the age pattern and intergroup-variation of the following smoking habit criteria were studied : per cent smokers, per cent pure cigarette smokers, per cent pure and mixed cigarette smokers, per cent heavy smokers, per cent heavy and medium smokers, per cent heavy pure cigarette smokers, per cent heavy and medium pure cigarette smokers. It was found that heavy smoking (more than 25 g. per day) is relatively infrequent in all groups and that cigarette smoking usually is most frequent in the younger age-classes. Female smokers—who for all practical purposes can be considered as pure cigarette smokers—are most frequent in the younger age-classes, whereas smoking among men is as frequent among the old as among the young. For most of the criteria studied the variation between the groups was considerable.

It was concluded that the variation found between the various groups indicates that great care should be taken in retrospective control studies designed to measure the association between tobacco smoking and lung cancer, to ensure correspondence between the control material and the lung cancer material with regard to sex, age, occupation and place of residence and that further studies are called for to find other factors associated with tobacco smoking. The present study may serve as a basis of comparison with the smoking habits of lung cancer patients, in the sense that findings generally outside the range of variation exhibited by the present material would be indicative of real deviations in the smoking habits of lung cancer patients, but quantitative statements about differences found cannot be inferred.

The low frequency of smokers in the higher age-classes among most groups of women may indicate that the increasing trend in total tobacco consumption per adult since 1928 partly may be caused by an increasing number of smokers in the population, rather than by increased consumption per smoker.

This study has been supported by a generous grant from " Tobaksfabrikernes Landsforening av 1901".

### REFERENCES.

DAFF, M. E., DOLL, R., AND KENNAWAY, E. L.—(1951) *Brit. J. Cancer*, **5**, 1.

KENNAWAY, E. L., AND KENNAWAY, N. M.—(1947) *Ibid.*, **1**, 260.

KORTEWEG, R.—(1953) *Docum. Med. Geogr. Trop.*, **5**, 2.

MILLS, C. A., AND PORTER, M. M. —(1953) *J. nat. Cancer Inst.*, **13**, 5.

### APPENDIX TABLES.

The separate groups are numbered throughout as in the list on p. 18.

*Explanation of Symbols.*

— No observation
.. Not applicable
( ) Percentage within brackets based on less than 15 observations.

### APPENDIX TABLE I.

| Year. | Yearly imports of tobacco leaf (tons). | Population (15 years and older). |
|---|---|---|
| 1900 | 1,865 | 1,452,687 |
| 1901 | 1,829 | — |
| 1902 | 1,843 | — |
| 1903 | 1,750 | — |
| 1904 | 1,295 | — |
| 1905 | 1,341 | — |
| 1906 | 1,582 | — |
| 1907 | 1,759 | — |
| 1908 | 1,655 | — |
| 1909 | 1,678 | — |
| 1910 | 1,879 | 1,557,938 |
| 1911 | 1,692 | — |
| 1912 | 1,976 | — |
| 1913 | 1,835 | — |
| 1914 | 2,107 | — |
| 1915 | 2,082 | — |
| 1916 | 2,346 | — |
| 1917 | 2,277 | — |
| 1918 | 1,550 | — |
| 1919 | 5,130 | — |
| 1920 | 3,115 | 1,802,370 |
| 1921 | 2,154 | — |
| 1922 | 2,374 | — |
| 1923 | 2,696 | — |
| 1924 | 2,475 | — |
| 1925 | 1,977 | — |
| 1926 | 2,260 | — |
| 1927 | 2,314 | — |

### Appendix Table II.

| Year. | Yearly imports of tobacco leaf (tons). | Quantity of tobacco smoked per year (tons). | Quantity of tobacco smoked per adult (g.). | Percentage distribution of quantity of tobacco smoked. Cigars. | Manufactured cigarettes | Hand-rolled cigarettes | Pipe tobacco. | Population (15 years and older). |
|---|---|---|---|---|---|---|---|---|
| 1928 | 2,363 | 1,630 | 828 | 4·47 | 34·71 | 0·25 | 60·57 | 1,967,662 |
| 1929 | 2,510 | 1,739 | 874 | 4·14 | 35·52 | 0·29 | 60·05 | 1,989,377 |
| 1930 | 2,476 | 1,902 | 945 | — | — | — | — | 2,013,308 |
| 1931 | 3,024 | 1,888 | 925 | — | — | — | — | 2,040,687 |
| 1932 | 2,286 | 1,883 | 909 | — | — | — | — | 2,070,933 |
| 1933 | 2,235 | 1,928 | 917 | — | — | — | — | 2,101,863 |
| 1934 | 2,451 | 2,026 | 952 | — | — | — | — | 2,128,511 |
| 1935 | 2,621 | 2,147 | 996 | — | — | — | — | 2,165,016 |
| 1936 | 2,821 | 2,334 | 1,063 | — | — | — | — | 2,195,675 |
| 1937 | 2,898 | 2,556 | 1,149 | 3·75 | 37·80 | 1·82 | 56·63 | 2,224,785 |
| 1938 | 2,869 | 2,625 | 1,165 | 3·77 | 36·81 | 2·27 | 57·15 | 2,253,642 |
| 1939 | 3,768 | 2,869 | 1,259 | 3·51 | 36·64 | 3·28 | 56·57 | 2,279,278 |
| 1940 | 2,463 | — | — | — | — | — | — | — |
| 1941 | 1,990 | — | — | — | — | — | — | — |
| 1942 | 1,939 | — | — | — | — | — | — | — |
| 1943 | 1,727 | — | — | — | — | — | — | — |
| 1944 | 428 | — | — | — | — | — | — | — |
| 1945 | 3,097 | 1,524 | 637 | — | — | — | — | 2,391,293 |
| 1946 | 4,341 | 3,732 | 1,514 | 0·89 | 40·94 | 9·65 | 48·52 | 2,465,450 |
| 1947 | 4,134 | 4,295 | 1,738 | 1·34 | 42·94 | 8·04 | 47·68 | 2,421,551 |
| 1948 | 4,063 | 4,125 | 1,666 | 1·32 | 40·48 | 6·57 | 51·63 | 2,475,480 |
| 1949 | 4,342 | 4,132 | 1,668 | 1·39 | 39·12 | 15·88 | 43·61 | 2,476,278 |
| 1950 | 3,735 | 4,001 | 1,616 | 1·20 | 37·77 | 18·37 | 42·66 | 2,476,000 |
| 1951 | 3,788 | 4,003 | 1,617 | 0·99 | 35·83 | 17·41 | 45·77 | 2,476,312 |

### Appendix Table III.—*Age Distribution. Males.*

| Group. | Age in years. 15–24. | 25–34. | 35–44. | 45–54. | 55–64. | 65–74. | 75–84. | 85–. | Not stated. | Total. |
|---|---|---|---|---|---|---|---|---|---|---|
| 1 | 22 | 27 | 65 | 62 | 36 | 13 | 1 | – | 5 | 231 |
| 2 | 97 | 199 | 165 | 133 | 78 | 23 | 1 | – | – | 696 |
| 3 | 38 | 116 | 127 | 82 | 64 | 10 | – | – | 3 | 440 |
| 4 | 9 | 583 | 785 | 605 | 207 | 78 | 62 | 8 | – | 2,337 |
| 5 | 63 | | 52 | 36 | 37 | 17 | 4 | – | – | 209 |
| 6 | 98 | 183 | 187 | 121 | 77 | 28 | 4 | 1 | – | 699 |
| 7 | 18 | 16 | 25 | 19 | 16 | 9 | 2 | – | – | 105 |

### Appendix Table IV.—*Age Distribution. Females.*

| Group. | Age in years. 15–24. | 25–34. | 35–44. | 45–54. | 55–64. | 65–74. | 75–84. | 85–. | Not stated. | Total. |
|---|---|---|---|---|---|---|---|---|---|---|
| 1 | 75 | 53 | 29 | 47 | 21 | 2 | – | – | 12 | 239 |
| 2 | 3 | 46 | 52 | 43 | 20 | 4 | – | – | – | 168 |
| 3 | 119 | | 51 | 38 | 46 | 29 | 6 | – | – | 289 |
| 4 | 56 | 53 | 72 | 61 | 40 | 15 | 4 | – | – | 301 |
| 5 | 2 | 9 | 13 | 16 | 8 | 2 | 2 | – | – | 52 |

### Appendix Table V.—*Per Cent Smokers. Males.*

| Group. | 15–24. | 25–34. | 35–44. | 45–54. | 55–64. | 65–74. | 75–84. | 85–. |
|---|---|---|---|---|---|---|---|---|
| | Age in years. | | | | | | | |
| 1 | 77·3 | 92·6 | 90·8 | 91·9 | 83·3 | (84·6) | (0·0) | .. |
| 2 | 72·2 | 92·5 | 94·5 | 91·0 | 80·8 | 78·3 | (100·0) | .. |
| 3 | 57·9 | 83·6 | 78·7 | 87·8 | 75·0 | (80·0) | .. | .. |
| 4 | (33·3) | 73·6 | 82·8 | 90·1 | 92·8 | 89·7 | 83·9 | (100·0) |
| 5 | 79·4 | | 86·5 | 86·1 | 81·1 | 70·6 | (100·0) | .. |
| 6 | 74·5 | 88·5 | 89·8 | 83·5 | 80·5 | 71·4 | (75·0) | (0·0) |
| 7 | 61·1 | 93·8 | 80·0 | 84·2 | 100·0 | (88·9) | (100·0) | .. |

### Appendix Table VI.—*Per Cent Pure Cigarette Smokers. Males.*

| Group. | 15–24. | 25–34. | 354–4. | 45–54. | 55–64. | 65–74. | 75–84. | 85–. |
|---|---|---|---|---|---|---|---|---|
| | Age in years. | | | | | | | |
| 1 | 54·5 | 63·0 | 60·0 | 58·1 | 36·1 | (23·1) | (0·0) | .. |
| 2 | 60·8 | 75·9 | 71·5 | 61·7 | 29·5 | 21·7 | (0·0) | .. |
| 3 | 36·8 | 28·4 | 26·0 | 23·2 | 14·1 | (10·0) | .. | .. |
| 4 | (22·2) | 47·9 | 52·1 | 50·7 | 34·8 | 20·5 | 22·6 | (0·0) |
| 5 | 47·6 | | 44·2 | 30·6 | 37·8 | 23·5 | (0·0) | .. |
| 6 | 53·1 | 53·0 | 59·9 | 48·8 | 32·5 | 14·3 | (0·0) | (0·0) |
| 7 | 38·9 | 43·8 | 40·0 | 63·2 | 50·0 | (22·2) | (0·0) | .. |

### Appendix Table VII.—*Per Cent Pure and Mixed Cigarette Smokers. Males.*

| Group. | 15–24. | 25–34. | 35–44. | 45–54. | 55–64. | 65–74. | 75–84. | 85–. |
|---|---|---|---|---|---|---|---|---|
| | Age in years. | | | | | | | |
| 1 | 63·6 | 70·4 | 70·8 | 62·9 | 44·4 | (23·1) | (0·0) | .. |
| 2 | 68·0 | 85·4 | 81·8 | 68·4 | 41·0 | 30·4 | (0·0) | .. |
| 3 | 55·3 | 47·4 | 39·4 | 36·6 | 26·6 | (10·0) | .. | .. |
| 4 | (33·3) | 67·4 | 69·4 | 71·4 | 53·6 | 44·9 | 30·6 | (0·0) |
| 5 | 55·6 | | 55·8 | 52·8 | 54·1 | 23·5 | (25·0) | .. |
| 6 | 72·4 | 81·4 | 81·8 | 66·1 | 54·5 | 25·0 | (25·0) | (0·0) |
| 7 | 50·0 | 68·8 | 68·0 | 78·9 | 56·2 | (33·3) | (0·0) | .. |

### Appendix Table VIII.—*Per Cent Heavy Smokers. Males.*

| Group. | 15–24. | 25–34. | 35–44. | 45–54. | 55–64. | 65–74. | 75–84. | 85–. |
|---|---|---|---|---|---|---|---|---|
| | Age in years. | | | | | | | |
| 1 | 4·5 | 3·7 | 7·7 | 3·2 | 2·8 | (0·0) | (0·0) | .. |
| 2 | 9·3 | 5·5 | 6·6 | 4·5 | 9·0 | 4·3 | (0·0) | .. |
| 3 | 0·0 | 0·0 | 0·8 | 1·2 | 0·0 | (0·0) | .. | .. |
| 4 | (0·0) | 3·3 | 5·7 | 8·4 | 6·8 | 10·3 | 0·0 | (0·0) |
| 5 | 4·8 | | 1·9 | 11·1 | 8·1 | 0·0 | (0·0) | .. |
| 6 | 6·1 | 3·8 | 7·5 | 7·4 | 3·9 | 17·9 | (25·0) | (0·0) |
| 7 | 5·6 | 0·0 | 0·0 | 10·5 | 6·3 | (11·1) | (0·0) | .. |

### Appendix Table IX.—*Per Cent Heavy and Medium Smokers. Males.*

| Group. | 15–24. | 25–34. | 35–44. | 45–54. | 55–64. | 65–74. | 75–84. | 85–. |
|---|---|---|---|---|---|---|---|---|
| | Age in years. | | | | | | | |
| 1 | 22·7 | 18·5 | 26·2 | 25·8 | 5·6 | (15·4) | (0·0) | .. |
| 2 | 15·5 | 20·6 | 28·5 | 30·1 | 21·8 | 13·0 | (0·0) | .. |
| 3 | 10·5 | 6·9 | 5·5 | 11·0 | 3·1 | (0·0) | .. | .. |
| 4 | (11·1) | 24·7 | 33·0 | 39·3 | 29·5 | 25·6 | 4·8 | (25·0) |
| 5 | 14·3 | | 17·3 | 27·8 | 24·3 | 0·0 | (25·0) | .. |
| 6 | 17·3 | 32·8 | 35·3 | 25·6 | 20·8 | 28·6 | (25·0) | (0·0) |
| 7 | 5·6 | 12·5 | 12·0 | 42·1 | 18·8 | (22·2) | (0·0) | .. |

### Appendix Table X.—*Per Cent Heavy Pure Cigarette Smokers. Males.*

| Group. | 15–24. | 25–34. | 35–44. | 45–54. | 55–64. | 65–74. | 75–84. | 85–. |
|---|---|---|---|---|---|---|---|---|
| | Age in years. | | | | | | | |
| 1 | 4·5 | 0·0 | 6·2 | 1·6 | 2·8 | (0·0) | (0·0) | .. |
| 2 | 3·1 | 3·5 | 4·2 | 2·3 | 5·1 | 0·0 | (0·0) | .. |
| 3 | 0·0 | 0·0 | 0·8 | 0·0 | 0·0 | (0·0) | .. | .. |
| 4 | (0·0) | 2·2 | 3·9 | 5·5 | 3·9 | 3·8 | 0·0 | (0·0) |
| 5 | 3·2 | | 0·0 | 8·3 | 2·7 | 0·0 | (0·0) | .. |
| 6 | 4·1 | 3·3 | 6·4 | 5·8 | 0·0 | 3·6 | (0·0) | (0·0) |
| 7 | 5·6 | 0·0 | 0·0 | 10·5 | 6·3 | (0·0) | (0·0) | .. |

### Appendix Table XI.—*Per Cent Heavy and Medium pure Cigarette Smokers. Males.*

| Group. | 15–24. | 25–34. | 35–44. | 45–54. | 55–64. | 65–74. | 75–84. | 85–. |
|---|---|---|---|---|---|---|---|---|
| | Age in years. | | | | | | | |
| 1 | 22·7 | 3·7 | 20·0 | 14·5 | 5·6 | (0·0) | (0·0) | .. |
| 2 | 8·2 | 16·6 | 22·4 | 21·1 | 10·3 | 0·0 | (0·0) | .. |
| 3 | 7·9 | 4·3 | 2·4 | 3·7 | 0·0 | (0·0) | .. | .. |
| 4 | (0·0) | 17·5 | 22·2 | 24·6 | 14·0 | 6·4 | 3·2 | (0·0) |
| 5 | 9·5 | | 9·6 | 13·9 | 5·4 | 0·0 | (0·0) | .. |
| 6 | 12·2 | 19·7 | 27·3 | 16·5 | 7·8 | 10·7 | (0·0) | (0·0) |
| 7 | 5·6 | 6·3 | 4·0 | 31·6 | 18·8 | (11·1) | (0·0) | .. |

### Appendix Table XII.—*Per Cent Smokers. Females.*

| Group. | 15–24. | 25–34. | 35–44. | 45–54. | 55–64. | 65–74. | 75–84. | 85–. |
|---|---|---|---|---|---|---|---|---|
| | Age in years. | | | | | | | |
| 1 | 48·0 | 66·0 | 34·5 | 31·9 | 0·0 | (0·0) | .. | .. |
| 2 | (33·3) | 40·0 | 67·3 | 46·5 | 50·0 | (50·0) | .. | .. |
| 3 | 20·2 | | 7·8 | 15·8 | 2·2 | 3·4 | (0·0) | .. |
| 4 | 50·0 | 49·1 | 43·1 | 27·9 | 12·5 | 6·7 | (50·0) | .. |
| 5 | (0·0) | (11·1) | (23·1) | 50·0 | (37·5) | (50·0) | (0·0) | .. |

### Appendix Table XIII.—*Per Cent Heavy Smokers. Females.*

| Group. | 15–24. | 25–34. | 35–44. | 45–54. | 55–64. | 65–74. | 75–84. | 85–. |
|---|---|---|---|---|---|---|---|---|
| | Age in years. | | | | | | | |
| 1 | 0·0 | 0·0 | 0·0 | 0·0 | 0·0 | (0·0) | .. | .. |
| 2 | (0·0) | 0·0 | 0·0 | 2·3 | 0·0 | (0·0) | .. | .. |
| 3 | 0·0 | | 0·0 | 0·0 | 0·0 | 0·0 | (0·0) | .. |
| 4 | 0·0 | 0·0 | 1·4 | 1·6 | 0·0 | 0·0 | (0·0) | .. |
| 5 | (0·0) | (0·0) | (0·0) | 0·0 | (0·0) | (0·0) | (0·0) | .. |

### Appendix Table XIV.—*Per Cent Heavy and Medium Smokers. Females.*

| Group. | 15–24. | 25–34. | 35–44. | 45–54. | 55–64. | 65–74. | 75–84. | 85–. |
|---|---|---|---|---|---|---|---|---|
| | Age in years. | | | | | | | |
| 1 | 2·7 | 1·9 | 0·0 | 0·0 | 0·0 | (0·0) | .. | .. |
| 2 | (0·0) | 8·7 | 13·5 | 11·6 | 5·0 | (0·0) | .. | .. |
| 3 | 0·8 | | 0·0 | 2·6 | 0·0 | 0·0 | (0·0) | .. |
| 4 | 7·1 | 9·4 | 9·7 | 3·3 | 5·0 | 0·0 | (0·0) | .. |
| 5 | (0·0) | (0·0) | (0·0) | 0·0 | (12·5) | (0·0) | (0·0) | .. |